# Association between ethylene oxide exposure and serum sex hormone levels measured in a reference sample of the US general population

**DOI:** 10.3389/fendo.2025.1533516

**Published:** 2025-04-11

**Authors:** Wenhao Wu, Jingna Wu, Zebin Hou, Qi Yan, Kaixin Qin, Yuan Zhao, Hua Zhang, Yikun Zhu, Junhua He, Jin Li

**Affiliations:** ^1^ Department of Endocrinology and Metabolism, The Second Hospital of Shanxi Medical University, Shanxi Medical University, Taiyuan, Shanxi, China; ^2^ Department of Thyroid Surgery, Shanxi Provincial People’s Hospital, Taiyuan, Shanxi, China

**Keywords:** ethylene oxide, endocrine disruptor, sex hormones, total testosterone, estradiol

## Abstract

Ethylene oxide (EO) is a crucial organic compound commonly utilized in industrial and medical products. Food and Drug Administration (FDA)-approved EO sterilization sterilizes about 50% of sterile medical devices in the U.S. Animal and human studies have suggested that EO exposure may result in severe health problem. However, studies evaluating the relationship between EO exposure and sex hormones in human populations are still lacking. Therefore, further investigation into EO’s effects on humans is essential. This cross-sectional study within the U.S. National Health and Nutrition Examination Survey (NHANES),2013–2016 examined the relationship between EO-hemoglobin adducts (HbEO) and sex hormones. HbEO was found to be inversely associated with estradiol (E2) and positively associated with the ratio of total testosterone (TT) to E2 and sex hormone-binding globulin (SHBG) levels in adult males. Such associations HbEO and E2 and SHBG were non-linear in male adults. However, no significant associations were found between HbEO and sex steroids across various age groups of females and all male age groups except for adults. Thus, our study provides evidence that EO may potentially serve as an endocrine disruptor in the environment, affecting the levels of sex hormones in adult males.

## 1 Introduction

Ethylene oxide (EO) is a crucial organic compound extensively employed in industrial and medical applications. It is extensively utilized as a disinfectant and sterilizing agent in numerous industrial processes to ensure product quality and safety (1). Additionally, it is employed in the manufacturing of various chemical products including ethylene glycol, emulsifiers, and surfactants (2). Despite its widespread applications, EO can cause potential hazards to human health. It exists in a gaseous form at room temperature, easily entering the respiratory system and bind to DNA and proteins. The blood biomarker N-(2-hydroxyethyl) valine, known as the hemoglobin adduct of EO (HbEO), is a valuable indicator of ethylene oxide exposure (3). *In-vitro* studies demonstrated that EO exposure exhibited genotoxic and mutagenic effects (4, 5). In population-based studies, it has been observed that long-term contact with EO may cause severe health issues such as cancer, cardiovascular diseases, diabetes, neurological impairments, respiratory diseases, as well as adverse reproductive outcomes (6–10). EO gas presents a significant health risk to residents, patients, and workers living in proximity to the currently hundreds of active EO-emitting facilities across the U.S. These individuals may be exposed to medical devices sterilized with EO within hospital environments, or directly as employees within EO-emitting manufacturing plants (11, 12). Although EO has previously been considered a “potentially hazardous air pollutant” in 2016, the U.S. Environmental Protection Agency (EPA) reclassified EO as a known human carcinogen based on new data, which indicate that the toxicity of EO is 30 times higher than previously estimated (5, 13). Given that there are a significant number of facilities emitting EO located in densely populated areas worldwide, understanding the comprehensive health risks associated with EO exposure is an urgent public health priority.

Sex hormones serve a crucial function in triggering and sustaining human reproductive well-being (14). Important human sex hormones include TT, E2, and SHBG play prominent roles (14).The levels of various sex hormones within the body vary at different stages of life. Studies on animals have demonstrated that exposure to EO by inhalation led to elongated estrous cycles in female mice (15), while male mice experienced testicular atrophy in the presence of EO exposure (16). These evidences implied detrimental impacts of EO on the reproductive system in mice. However, currently, studies in human populations evaluating the correlation between exposure to EO and sex steroid hormone are still lacking. Hence, the objective of this research is to investigate the connection between exposure to EO and sex hormones across various demographic segments of the human population.

## 2 Materials and methods

### 2.1 Study design and population

**Figure 1:**
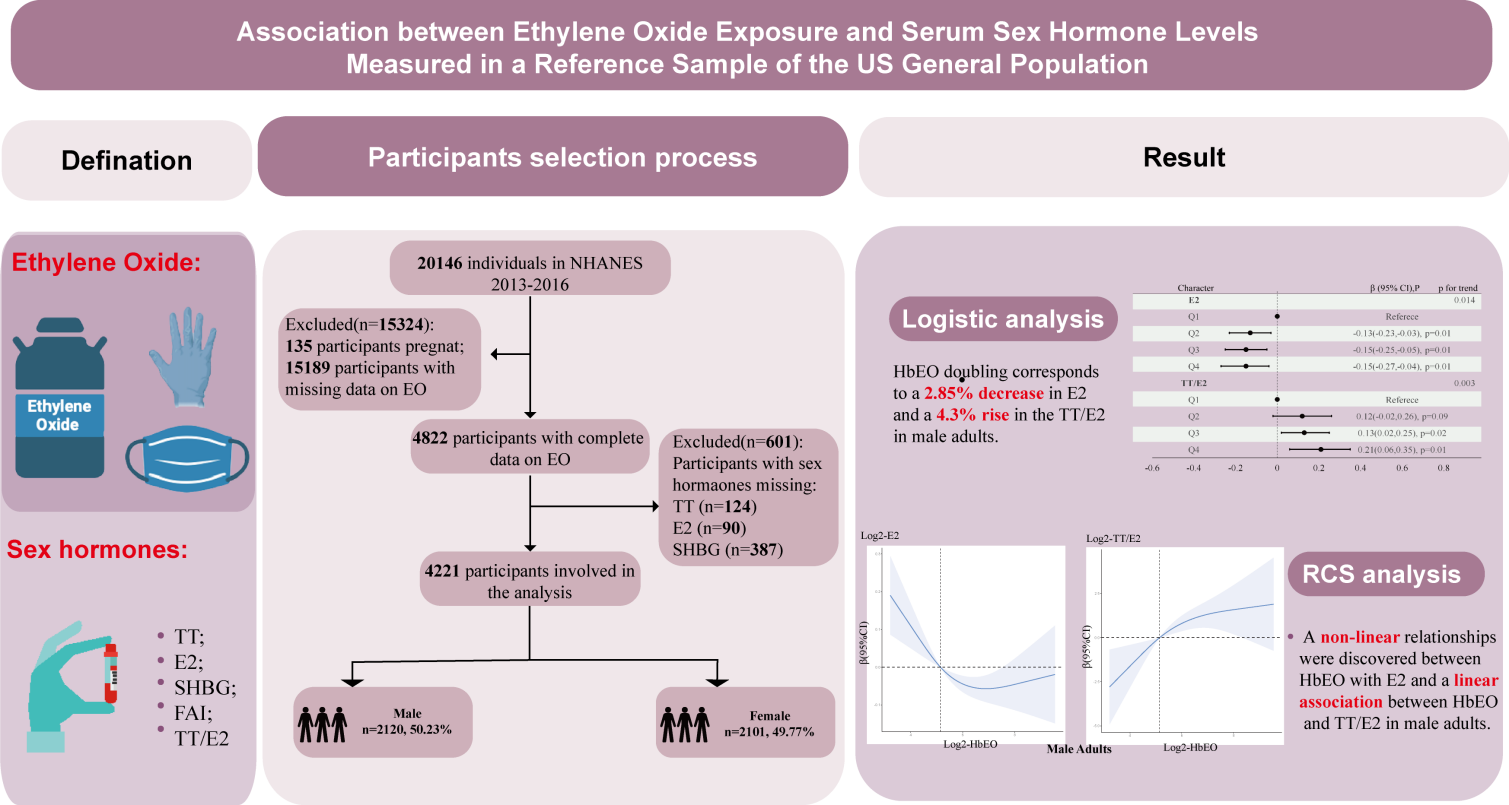
Graphical abstract and flowchart of studied participants selection. TT, total testosterone; E2, total estradiol; SHBG, sex hormone binding globulin; FAI, free androgen index, was calculated as total testosterone (ng/dL)/SHBG (nmol/L), TT/E2 was calculated as TT (ng/dL)/E2 (pm/ml); HbEO, hemoglobin adducts of ethylene oxide.

NHANES survey measures the health and nutrition of American adults, adolescents and children in the most comprehensive way. This survey combines information from interviews and physical examinations, which information from interviews and physical examinations is combined. NHANES is conducted under the National Center for Health Statistics (NCHS), an agency that falls under the jurisdiction of the Centers for Disease Control and Prevention (17).Data collection and laboratory analysis methods for the NHANES are described elsewhere. Questions on demographics, socioeconomics, dietary habits, and health are included in the interviews. Participants gave written consent, and the study’s methods and materials were ethics board-approved. This study used data from the 2013–2016 NHANES to determine blood HbEO, serum TT, E2, and SHBG levels for the United States population. Referring to previous NHANES literature, we deleted participants with missing covariates (18, 19). A total of 20146 participants were enrolled at first, after the exclusion of individuals who were pregnant (*n*=135), missed data on blood EO (*n*=15189) and unavailable data on sex hormones (n=601), 4221 participants were included in our final analysis. **Figure 1** illustrates the selection process for participants.

### 2.2 Measurements of blood ethylene oxide

HbEO, with a longer *in vivo* half-life than EO, is utilized as an indicator of EO exposure. The measurement of HbEO levels was performed according to the guidelines outlined in the NHANES Laboratory/Medical Technologist Procedures Manual (https://wwwn.cdc.gov/Nchs/Nhanes/2013-2014/ETHOX_H.htm). Initially, samples of erythrocytes were gathered, preserved at −30°C, and then sent to the National Center for Environmental Health for assessment. Subsequently, The HbEO quantity was measured utilizing high-performance liquid chromatograph-tandem mass spectrometry (HPLC-MS/MS) and the modified Edman reaction. Lastly, as a result, the levels of HbEO were expressed as picomoles adducts per gram of hemoglobin. Additionally, a comprehensive quality control program, incorporating both external and internal surveillance, was established to oversee and assess the accuracy and reliability of the analytical testing process.

### 2.3 Sex hormone measurement

A serum sample was prepared, preserved at -20°C, and shipped to the National Center for Environmental Health to be tested. To measure TT and E2, isotope dilution-liquid chromatograph-tandem mass spectrometry (ID-LC-MS/MS) was applied. The levels of SHBG were measured using a chemiluminescent assay. Detailed technical information about these methods are available in other sources (20, 21). The lower limit of detection (LLOD) was 0.75 ng/mL for TT, 2.994 pg/mL for E2, and 0.800 nmol/L for SHBG. For estimating the concentration of circulating free testosterone, the free androgen index (FAI) was used to estimate the level of circulating free testosterone by dividing TT by SHBG (22). The TT to E2 ratio (TT/E2) was utilized as an indirect measure of aromatase activity (23).

### 2.4 Other covariates

Potential confounding factors were also collected as covariates, including age, race/ethnicity, education level, BMI, poverty income ratio (PIR), cotinine and the time of sample collection. Race/ethnicity was classified into Hispanics, non-Hispanic Black, non-Hispanic White, and other races. Education level was classified as less than high school, more than high school, high school, or general educational development (24). BMI was computed by dividing weight (in kilograms) by the square of height (in meters). According to the International Obesity Task Force (IOTF), children were classified as overweight (BMI ≥ IOTF-25), obesity (BMI ≥ IOTF-30) and normal (BMI< IOTF-25) (25). In the adult group, BMI category was classified as < 25 kg/m2 and ≥ 25 kg/m2 (9). The PIR, which signifies the family’s collective socioeconomic standing, was computed by dividing the family income according to the poverty guidelines pertinent to the participant’s household size, coupled with the year and state data (26). Serum cotinine levels were measured and group into 2 categories: <LOD (below the LOD, 0.015 ng/mL) and ≥LOD to take the environmental tobacco exposure into account (27). Moreover, to account for the variations in hormone levels, a six-month time span (November 1 to April 30, May 1 to October 31) incorporated as covariates in the statistical analysis. Physical activity was calculated based on a detailed physical activity survey described previously (28). Total energy intake was calculated from three-day dietary-recall food composition tables (29).Whether the participant has taken prescription medicine is assessed by NHANES interviewers through the following question: “In the past 30 days, have you used or taken any medication that requires a prescription? Please do not include any prescription vitamins or minerals that you may have already mentioned.” according to the NHANES analysis guideline (https://wwwn.cdc.gov/Nchs/Data/Nhanes/Public/2013/DataFiles/RXQ_RX_H.htm#RXDCOUNT).

Potential confounding factors were also collected as covariates, including age, race/ethnicity, education level, BMI, poverty income ratio (PIR), cotinine and the time of sample collection. Race/ethnicity was classified into Hispanics, non-Hispanic Black, non-Hispanic White, and other races. Education level was classified as less than high school, more than high school, high school, or general educational development (24). BMI was computed by dividing weight (in kilograms) by the square of height (in meters). According to the International Obesity Task Force (IOTF), children were classified as overweight (BMI ≥ IOTF-25), obesity (BMI ≥ IOTF-30) and normal (BMI< IOTF-25) (25). In the adult group, BMI category was classified as < 25 kg/m2 and ≥ 25 kg/m2 (9). The PIR, which signifies the family’s collective socioeconomic standing, was computed by dividing the family income according to the poverty guidelines pertinent to the participant’s household size, coupled with the year and state data (26). Serum cotinine levels were measured and group into 2 categories: <LOD (below the LOD, 0.015 ng/mL) and ≥LOD to take the environmental tobacco exposure into account (27). Moreover, to account for the variations in hormone levels, a six-month time span (November 1 to April 30, May 1 to October 31) incorporated as covariates in the statistical analysis. Physical activity was calculated based on a detailed physical activity survey described previously (28). Total energy intake was calculated from three-day dietary-recall food composition tables (29). Whether the participant has taken prescription medicine is assessed by NHANES interviewers through the following question: “In the past 30 days, have you used or taken any medication that requires a prescription? Please do not include any prescription vitamins or minerals that you may have already mentioned.” According to the NHANES analysis guideline (https://wwwn.cdc.gov/Nchs/Data/Nhanes/Public/2013/DataFiles/RXQ_RX_H.htm#RXDCOUNT). Urine specimens were obtained during field examination visits and cryopreserved at −20°C prior to analytical processing. Chemical selection criteria required ≥85% detection frequency across samples, resulting in the inclusion of: two phenolic compounds (bisphenol A [BPA] and bisphenol S [BPS]), three phthalate derivatives (monobenzyl phthalate [MBzP], monoisobutyl phthalate [MiBP], and monocarboxyoctyl phthalate [MCOP]). BPA and BPS were quantified via automated online solid-phase extraction coupled with isotope-dilution high-performance liquid chromatography-tandem mass spectrometry (SPE-HPLC-MS/MS). MBzP, MCOP, and MiBP analysis employed HPLC separation with electrospray ionization tandem mass spectrometric detection (HPLC-ESI-MS/MS). Methodological specifications and quality assurance protocols adhere to NHANES laboratory standards (https://wwwn.cdc.gov/Nchs/Data/Nhanes/Public/2013/DataFiles/EPHPP_H.htm#URD14DLC), (https://wwwn.cdc.gov/Nchs/Data/Nhanes/Public/2013/DataFiles/PHTHTE_H.htm), polychlorinated biphenyls (PCBs) were measured in serum by high-resolution gas chromatography/isotope-dilution high-resolution mass spectrometry (https://wwwn.cdc.gov/Nchs/Data/Nhanes/Public/2013/DataFiles/PCBPOL_H.htm). Based on prior literature, we identified three polychlorinated biphenyls (PCBs) with potential endocrine-disrupting effects on sex hormones (30).

### 2.5 Statistical analysis

Appropriate weighting, in accordance with the recommendations of the NCHS, were employed for each analytical process. Weighted mean value (± standard deviation [SD]) were calculated employing NHANES primary sampling units and strata, with the results being subjected to a weighted t-test for statistical analysis. Frequencies (proportions) for categorical variables were analyzed using the weighted chi-square test. The serum HbEO and sex hormone indicator distributions exhibited a right-skewed pattern, prompting log2 transformation to normalize them for descriptive and regression analyses. The log2-transformed HbEO values were then analyzed as continuous and categorical (divided into quartiles) variables (31). Weighted quartiles for log2-transformed HbEO were calculated within specific sex-age and sex-puberty subgroups, as shown in **Supplementary Table S1**.

A weighted multiple linear regression was used to calculate β (Standardized coefficients) values and corresponding 95% confidence intervals (CIs) to examine the connections between individual HbEO levels and sex steroid hormone indicators. Restricted cubic spline (RCS) analysis was applied to further examine linear and non-linear relationships between HbEO and sex hormones after adjusting for various potential covariates (32, 33). The Akaike information criterion (AIC) was used to choose the most suitable knots that had the smallest AIC (34).

Considering the marked variations in sex hormone levels between genders and at different stages of development, this analysis was conducted based on age (children: ≤11 years, adolescents: 12-19 years, adults: >19 years) for males and females (35). Grouping 6-19 years old participants as “children” or “adolescents” based solely on their ages may include both pre-pubertal and pubertal individuals in the same groups. Such grouping could result in subgroups with exceptionally high or low sex hormone levels, which might bias the regression analysis relationship between HbEO and sex steroid hormones. Moreover, the impact of log2-HbEO on sex hormones may be influenced by the pubertal stage. To tackle this concern, we further subcategorized individuals into pubertal and prepubertal subgroups based on their serum sex hormone levels and menarcheal status. Individuals were considered to have entered puberty (i.e., classified as pubertal) if their TT levels were equal to or greater than 50 ng/dL (in males) or E2 levels were 20 pg/mL or higher (in females), or if they had experienced the onset of menstruation (in females) (36–38). Those not meeting these criteria were classified as pre-pubertal (i.e., classified as prepubertal). Data regarding girls’ menarche status was collected through inquiries such as: “Have menstrual periods commenced?” (found in the medical questionnaire) and “What age was the first menstrual period?” (included in the reproductive health questionnaire). Female whose answer was “Yes” or provided their age at first menstrual period was recorded as having started menstrual cycles. Furthermore, this analysis performed the multiple linear regression analyses again, stratified by pubertal status in both males and females.

Subgroup analyses with multiplicative interaction terms were performed to show whether the relationship between individual HbEO levels and sex steroid hormones varied by race, age (<45 years or ≥45 years), BMI (<24 or ≥24 kg/m2), energy intake (<2,400 or ≥ 2,400 kcal), physical activity (<200 or ≥ 200 METs-hour/week) and taken prescription medicine (Yes or No) (39).

To evaluate the robustness of our results, 4 sensitivity analyses were conducted to examine the relationship between individual HbEO levels and sex steroid hormones: 1) Multiple imputation for missing data was conducted using the ‘mice’ package (Multivariate Imputation by Chained Equations) in R (40); 2) Energy intake, physical activity, and prescription medication was adjusted additionally; 3) exposure of bisphenol A, phthalates and polychlorinated biphenyls was adjusted additionally; 4) excluded participants aged ≥65 years in the adults. All statistical analyses were performed using R software version 4.1.2, and a two-tailed P value with a significance level of 0.05 was used for all statistical tests to determine significance.

## 3 Results

### 3.1 Descriptive statistics

**Table 1** summarizes the demographic features of the enrolled participants categorized by sex and age. The study sample comprised a total of 4221 individuals, including 2089 males and 2101 females. The individuals had a mean age of 41.70 years. The detection rates for TT, E2, SHBG, and HbEO in the entire sample were 99.81%, 85.93%, 100%, and 97.49%, respectively. The values below the LLOD were replaced by the lower limit of detection divided by the square root of 2 (LLOD/sqrt (2)) according to the NHANES analysis guideline (https://wwwn.cdc.gov/Nchs/Data/Nhanes/Public/2013/DataFiles/ETHOX_H.htm#LBXEOA) (**Supplementary Table S2**). However, the detection rate for E2 in male children was less than 50%. As a result, the analyses for E2 and TT/E2 were omitted for this particular subgroup. Comparatively, adult participants exhibited greater ethylene oxide exposure levels than adolescents, while adolescents showed higher exposure levels than children, across both genders (**Supplementary Table S3**).

**Table 1:** Weighted sample characteristics of children (6-11 years old), adolescents (12–19 years old) and adults (≥ 20 years old) with serum sex hormones and HbEO in NHANES 2013–2014.

Variable	ALL	Female					Male				
		ALL	Children	Adolescents	Adults	P-value	ALL	Children	Adolescents	Adults	P-value
**Age** ^a^	41.70 (0.46)	42.58 (0.60)	8.63 (0.17)	15.39 (0.17)	48.68 (0.66)	< 0.0001	40.82 (0.55)	8.49 (0.11)	15.24 (0.14)	46.88 (0.60)	< 0.0001
**BMI** ^a^	28.05 (0.20)	28.61 (0.28)	18.80 (0.40)	25.13 (0.46)	29.80 (0.32)	< 0.0001	27.50 (0.18)	18.80 (0.37)	23.13 (0.41)	28.80 (0.18)	< 0.0001
**Race/ethnicity** ^b^						< 0.001					< 0.0001
White (Non-Hispanic)	1478 (63.08)	773 (63.10)	98 (56.04)	603 (64.99)	72 (51.54)		705 (63.06)	72 (50.56)	574 (65.74)	59 (49.03)	
Non-Hispanic Black	828 (10.88)	438 (10.54)	75 (12.57)	312 (10.19)	51 (11.59)		390 (11.21)	64 (12.64)	271 (10.98)	55 (11.88)	
Hispanics	772 (10.56)	352 (10.47)	64 (14.85)	231 (9.41)	57 (16.27)		420 (10.64)	90 (15.91)	260 (9.26)	70 (19.83)	
Other	1143 (15.49)	557 (15.88)	83 (16.53)	395 (15.41)	79 (20.59)		586 (15.09)	110 (20.89)	405 (14.03)	71 (19.25)	
**PIR** ^a^	2.87 (0.08)	2.78 (0.10)	2.34 (0.24)	2.20 (0.13)	2.89 (0.09)	< 0.0001	2.95 (0.09)	2.41 (0.17)	2.45 (0.16)	3.06 (0.08)	< 0.0001
**Serum cotinine (ng/mL)** ^a^	50.74 (3.65)	32.62 (2.75)	0.37 (0.08)	5.41 (3.44)	38.59 (3.22)	< 0.0001	68.74 (5.60)	0.27 (0.06)	19.03 (6.43)	80.96 (6.71)	< 0.0001
**Cotinine exposure status** ^b^						0.61					0.57
Exposed (> 0.015 ng/ml)	2844 (67.43)	1320 (61.25)	155 (57.68)	197 (60.23)	968 (61.66)		1524 (69.77)	173 (66.08)	231 (71.79)	1120 (69.80)	
Unexposed (≤0.015 ng/ml)	1374 (32.57)	780 (38.75)	100 (42.32)	139 (39.77)	541 (38.34)		594 (30.23)	85 (33.92)	89 (28.21)	420 (30.20)	
**Six month time period** ^b^						0.95					0.99
May 1 through October 31	2136 (50.6)	1112 (57.88)	134 (59.35)	180 (58.20)	798 (57.73)		1024 (52.98)	131 (53.49)	150 (52.34)	743 (53.02)	
November 1 through April 30	2085 (49.4)	989 (42.12)	121 (40.65)	156 (41.80)	712 (42.27)		1096 (47.02)	128 (46.51)	170 (47.66)	798 (46.98)	
**Education level** ^b^						< 0.0001					< 0.0001
High school or general educational development	1372 (32.51)	652 (29.32)	0 (0.00)	174 (55.36)	478 (28.28)		720 (32.09)	0 (0.00)	154 (45.74)	566 (32.89)	
Less than high school	1140 (27.01)	566 (15.57)	255 (100.00)	149 (42.40)	162 (5.61)		574 (17.14)	259 (100.00)	147 (48.90)	168 (6.12)	
More than high school	1708 (40.47)	883 (55.10)	0 (0.00)	13 (2.25)	870 (66.11)		825 (50.77)	0 (0.00)	19 (5.36)	806 (60.98)	
**Energy intake (kcal/d)** ^b^						0.11					< 0.0001
<2400	2639 (61.48)	1511 (73.20)	178 (85.80)	255 (80.12)	1078 (78.97)		1128 (49.82)	160 (72.33)	187 (62.62)	781 (50.55)	
≥2400	1187 (31.36)	378 (18.89)	34 (14.20)	64 (19.88)	280 (21.03)		809 (43.76)	53 (27.67)	118 (37.38)	638 (49.45)	
**Physical activity (METs-hour/week)** ^b^						0.76					0.59
<200	130 (2.98)	66 (3.28)	0 (NA)	14 (3.54)	52 (4.86)		64 (2.68)	0 (NA)	10 (2.02)	54 (3.70)	
≥200	2691 (70.42)	1259 (66.62)	0 (NA)	266 (96.46)	993 (95.14)		1432 (74.21)	0 (NA)	277 (97.98)	1155 (96.30)	
**Prescription medicine** ^b^						< 0.0001					< 0.0001
No	2220 (46.68)	1035 (40.98)	221 (86.60)	263 (74.29)	551 (33.21)		1185 (52.34)	187 (67.38)	253 (76.50)	745 (47.90)	
Yes	1999 (53.30)	1065 (59.01)	34 (13.40)	72 (25.71)	959 (66.79)		934 (47.63)	72 (32.62)	67 (23.50)	795 (52.10)	
**Serum sex hormones indices** ^a^											
TT (ng/dL)	205.25 (5.05)	23.00 (0.45)	8.55 (0.69)	27.16 (0.89)	23.59 (0.57)	< 0.0001	386.42 (5.93)	17.54 (4.64)	390.04 (17.38)	416.08 (5.64)	< 0.0001
SHBG (nmol/L)	61.18 (1.25)	75.74 (1.97)	88.19 (4.57)	67.34 (4.43)	75.85 (2.05)	0.01	46.70 (0.87)	106.07 (5.14)	42.23 (1.36)	42.45 (0.62)	< 0.0001
E2 (pg/mL)	40.40 (1.40)	58.72 (2.67)	14.19 (2.11)	77.90 (5.01)	59.73 (3.13)	< 0.0001	22.19 (0.33)	2.47 (0.12)	19.09 (0.74)	24.22 (0.36)	< 0.0001
FAI	5.58 (0.14)	0.42 (0.01)	0.17 (0.02)	0.62 (0.03)	0.42 (0.01)	< 0.0001	10.71 (0.18)	0.34 (0.09)	12.06 (0.50)	11.38 (0.20)	< 0.0001
TT/E2	10.07 (0.30)	1.98 (0.08)	1.44 (0.15)	0.86 (0.16)	2.16 (0.09)	< 0.0001	18.12 (0.46)	4.21 (0.55)	22.12 (0.81)	18.72 (0.49)	< 0.0001
**HbEO (pmol/gHb)** ^a^	76.31 (3.59)	69.06 (4.96)	36.09 (1.26)	39.41 (5.69)	75.40 (5.62)	< 0.0001	83.52 (5.21)	36.15 (1.55)	52.89 (6.97)	91.48 (5.96)	< 0.0001

a Weighted mean value (± standard deviation [SD]).

b Frequencies (proportions) as appropriate.

PIR, the ratio of family income to poverty, was calculated by dividing family income by the poverty guidelines specific to family size, as well as the appropriate year and state. TT, total testosterone; E2, total estradiol; SHBG, sex hormone binding globulin; FAI, free androgen index, was calculated as total testosterone (ng/dL)/SHBG (nmol/L), TT/E2 was calculated as TT (ng/dL)/E2 (pm/ml). BMI, body mass index; HbEO, hemoglobin adducts of ethylene oxide.

Given the crucial involvement of sex hormones in the initiation of puberty, the statuses of pre-puberty and puberty were also examined (**Supplementary Table S4**). The proportion of E2 detections in prepubertal males and females was below 50%. Consequently, assessments of E2 and TT/E2 were not conducted for these demographic subsets. EO exposure was significantly higher in the puberty than that of pre-puberty in males. While there were no significances between pre-puberty and puberty in females.

### 3.2 The associations between individual HbEO levels and sex hormone levels across sex-age and sex-puberty categories

**Figure 2:**
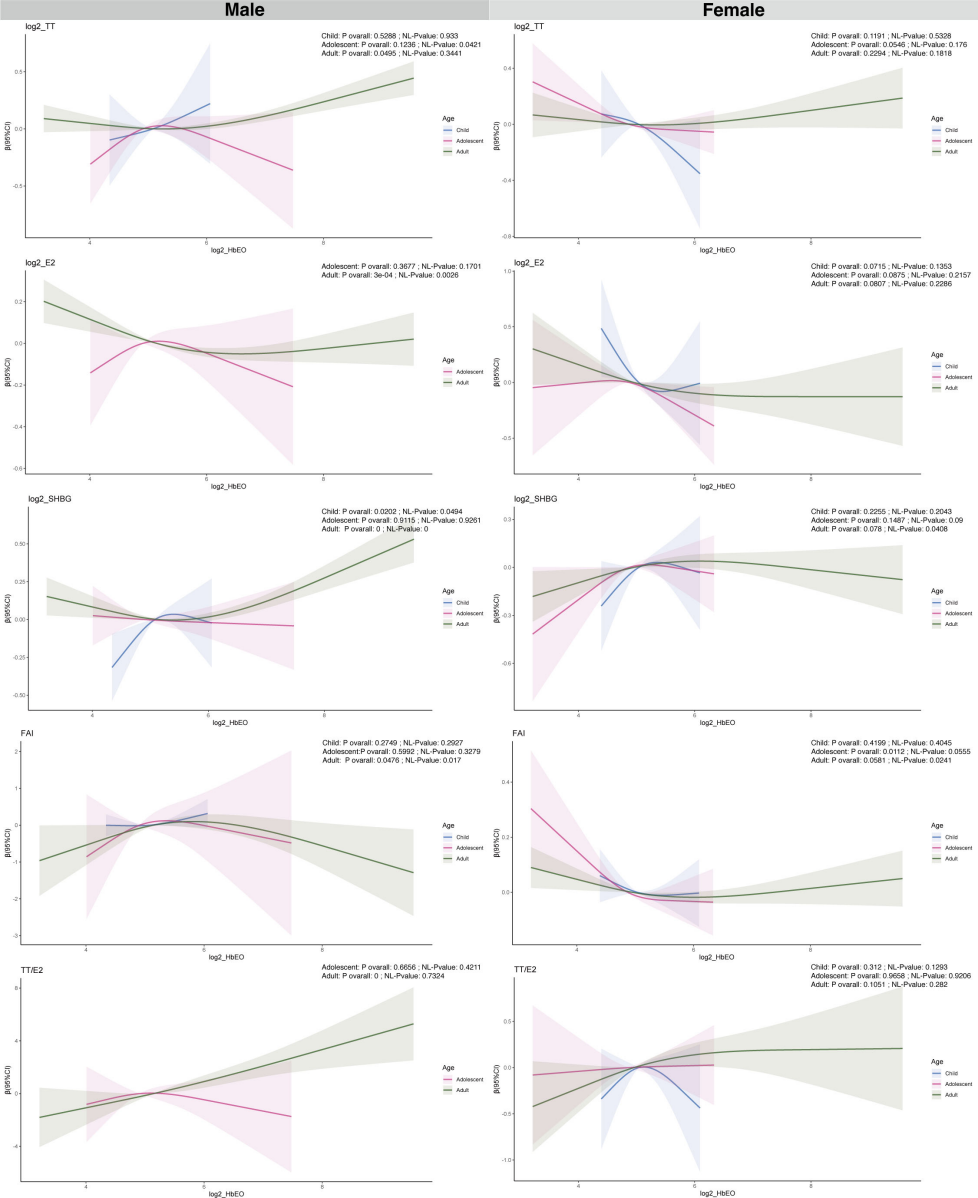
Analysis of the association between log2-HbEO and sex hormones by sex-age groups in participants in NHANES 2013–2016 using the RCS. TT, total testosterone; E2, total estradiol; SHBG, sex hormone binding globulin; FAI, free androgen index, was calculated as total testosterone (ng/dL)/SHBG (nmol/L), TT/E2 was calculated as TT (ng/dL)/E2 (pm/ml). HbEO, hemoglobin adducts of ethylene oxide.

**Figure 2** and **Supplementary Table S4** present the relationships between HbEO levels, categorized as continuous and quartiles, and sex hormones across various stages of development. Among males, no substantial correlations were found between HbEO and sex steroid hormones among children and adolescents (**Supplementary Table S4**). However, in male adults, HbEO doubling corresponds to a 2.85% decrease in E2, a 2.10% increase in SHBG, and a 4.3% rise in the TT/E2 (for E2: p= 0.04, OR = -0.03, 95% CI: -0.06 to 0.00; for SHBG: p= 0.03, OR = 0.03, 95% CI: 0.00 to 0.06; for TT/E2: p< 0.001, OR = 0.06, 95% CI: 0.03 to 0.09). Moreover, consistent associations of serum E2 and TT/E2 levels with HbEO were also observed across quartiles of EO exposure. Serum E2 levels were decreased along with the increased exposure of EO (p for trend=0.014). Similarly, TT/E2 levels were increased accompanied by the increased exposure of EO (p for trend=0.003) (**Figure 2**). No correlations were identified between EO exposure and sex hormones in female subjects across various categories.

The relationship between the continuous and quartile distributions of HbEO and sex steroid hormones in prepubertal and pubertal status were presented in **Supplementary Table S5**. There were no significant correlations found between HbEO and sex steroid hormones in prepuberty and puberty, irrespective of gender. When evaluated by quartiles of EO exposure, HbEO (quartile 4 versus 1) showed a positive correlation with SHBG in pre-pubertal males (P = 0.03, OR = 0.21, 95% CI: 0.02, 0.4). In contrast, HbEO (quartile 3 versus 1) was negatively correlation with SHBG in pubertal males (P = 0.03, OR = -0.22, 95% CI: -0.42, -0.03).

### 3.3 Association between individual log2_HbEO and sex steroid hormone indicators by sex-age and sex-puberty groups shown by RCS

**Figure 3:**
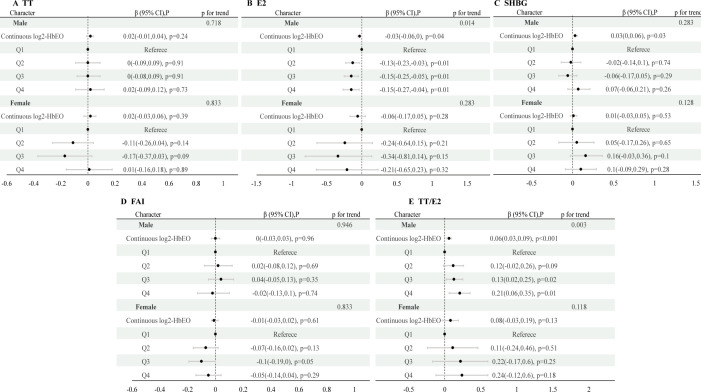
Associations of continuous and quartiles of log2-HbEO with sex hormones in >19-year old adults. **(A)** TT. **(B)** E2. **(C)** SHBG. **(D)** FAI. **(E)** TT/E2. TT, total testosterone; E2, total estradiol; SHBG, sex hormone binding globulin; FAI, free androgen index, was calculated as total testosterone (ng/dL)/SHBG (nmol/L), TT/E2 was calculated as TT (ng/dL)/E2 (pm/ml); HbEO, hemoglobin adducts of ethylene oxide.

A restricted cubic spline (RCS) analysis was utilized for further investigating the linear and nonlinear relationships between HbEO with sex hormones after adjusting for various confounding factors, as depicted in **Figure 3** and **Supplementary Figure S1**. In male children, A nonlinear association was found between HbEO and SHBG (NL-P value = 0.049). A non-linear relationship was found between HbEO and TT in male adolescents (NL-P value = 0.042). In male adult, non-linear relationships were discovered between HbEO with E2, SHBG and FAI (E2: NL-P value = 0.0026; SHBG: NL-P value =0; FAI: NL-P value =0.017). Furthermore, nonlinear associations were found between FAI, SHBG, and HbEO in female adult participants. (NL-P value = 0.041; FAI: NL-P value = 0.024) (**Figure 3**).

What’s more, our RCS analysis uncovered a nonlinear relationship between HbEO and SHBG in the prepubertal group among males (NL-P value = 0.0264). While, HbEO has a non-linear association with FAI in female pubertal individuals (NL-P value = 0.0476) (**Supplementary Figure S1**).

### 3.4 Subgroup analysis and sensitive analysis

**Figure 4:**
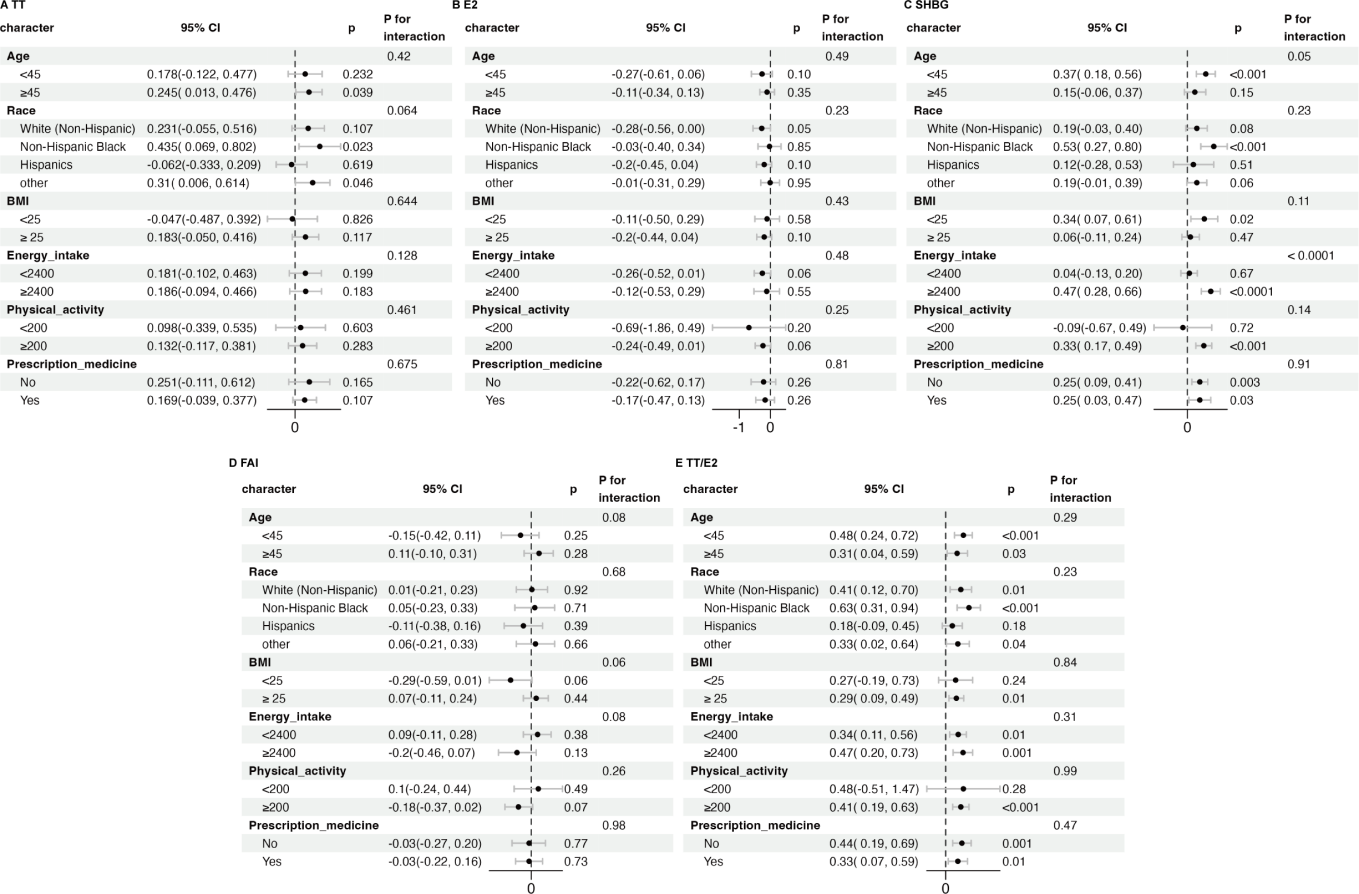
Subgroup analysis of the association between log2-HbEO and sex hormones in subgroups of >19-year old male adults. **(A)** TT. **(B)** E2. **(C)** SHBG. **(D)** FAI. **(E)** TT/E2. Each subgroup adjusted for all factors except the stratification factor itself. TT, total testosterone; E2, total estradiol; SHBG, sex hormone binding globulin; FAI, free androgen index, was calculated as total testosterone (ng/dL)/SHBG (nmol/L), TT/E2 was calculated as TT (ng/dL)/E2 (pm/ml). BMI, body mass index; HbEO, hemoglobin adducts of ethylene oxide.

Subgroup analyses were conducted among adult males categorized by age, race, BMI, energy intake, physical activity, and prescription medication to further investigate the consistency of the relationship between the log2-transformed HbEO levels and sex hormones across different groups. These results confirmed a robust association between the log2-transformed HbEO levels and TT, E2, FAI, TT/E2 across different subgroups (P for interaction > 0.05) (**Figures 4A, B, D, E**). We found that the effect of HbEO on SHBG was significantly dependent on energy intake (interaction P < 0.001). In adult males with an energy intake ≥ 2400 kcal, HbEO was positively associated with SHBG (**Figure 4C**).

To validate the robustness of our findings, we conducted 4 sensitivity analyses. In all sensitivity analyses, the negative association of HbEO with E2 and TT/E2 remained significant, demonstrating the robustness of our results (**Supplementary Tables S6–S12**).

## 4 Discussion

This investigation represents the first to explore the connection between EO and sex steroids across various age brackets within a participant population. The findings demonstrated that HbEO was significantly inversely associated with E2 and positively associated with TT/E2 and SHBG levels in adult males. Such associations between HbEO with E2 and SHBG were non-linear in male adults. However, no significant correlation was detected between HbEO and sex steroids in different age female groups. Thus, our study provides evidence that EO may potentially serve as an endocrine disruptor in the environment, affecting the levels of sex hormones in adult males.

EO is a gaseous compound at room temperature and is primarily encountered through inhalation (41). Members of the public may encounter EO through a range of routes, such as breathing polluted air, inhaling tobacco smoke, exposure to vehicle exhaust, or encountering fumes from commercial products in domestic settings (1, 42). a key industrial chemical derived from ethylene, is extensively employed in the manufacturing of diverse compounds that are prevalent in industrial, medical, and consumer goods (4). Nevertheless, in 2016, Ethylene oxide is classified as a carcinogen for humans by both the International Agency for Research on Cancer (IARC) and the United States Environmental Protection Agency (USEPA), signifying that ethylene oxide is harmful to human health’ sake (43). EO sterilization, FDA, is responsible for sterilizing approximately half of all sterile medical devices within the U.S. Workers, especially those in the medical equipment sterilization sector, have been documented to face occupational exposure to EO at elevated levels (2, 44). EO sterilization has also gained great attention in the worldwide effort to combat the COVID-19 pandemic. The rise in requests for personal protective equipment (PPE), including items like face masks, gloves, and protective suits, might have led to a higher exposure to EO. It is noteworthy that single-use sterile medical devices have stringent standard regarding EO residue. Specifically, the allowable residual limit for EO limit content in face masks is established at <10 μg/g. These regulatory standards underscore the importance of conducting interventions limiting the exposure of EO in daily life. HbEO serve as a more stable and sensitive blood biomarker for assessing EO exposure. Prior research has established a direct link between human HbEO levels and exposure to airborne EO (45, 46). In this investigation, we utilized a widely recognized HPLC-MS/MS that integrates a modified Edelman reaction to concurrently measure HbEO level (47). Occupational exposure limits for EO are set by organizations such as the Occupational Safety and Health Administration (OSHA) in the United States. The current OSHA permissible exposure limit (PEL) for EO is 1 ppm (parts per million) as an 8-hour time-weighted average. OSHA regulations require employers to implement controls to reduce EO exposure below the PEL (48). The U.S. Food and Drug Administration (FDA) regulates the use of EO in the sterilization of medical devices and sets limits on the residual amounts of EO left on these devices (49). The variability in EO regulations across different countries and states can lead to inconsistencies in protection levels. There can be challenges in enforcing regulations due to the pervasive use of EO and the difficulty of monitoring and controlling emissions. Workers in industries that use EO, such as healthcare workers and those in the sterilization and chemical manufacturing industries, are at highest risk. Communities located near facilities that emit EO may be at increased risk of exposure. Ensuring compliance with exposure limits requires effective monitoring and enforcement, which can be resource-intensive. Public awareness about the risks of EO exposure is crucial for informed decision-making and for the public to advocate for stronger protections. As more is learned about the health effects of EO, exposure limits may need to be revised and additional controls implemented to protect public health. There may be a need for enhanced research into the health effects of EO and improved methods for monitoring and controlling its emissions.

Only a few animal experiments have explored the relationship between HbEO and sex steroid. Previous studies have found that chronic inhalation of EO leads to a dose-dependent decrease in testes weight and testes’ DNA content in adult male rats (50). Some studies have also found damages to reproductive cells and impaired sperm production in male rats after EO exposure (15, 51). In human studies, Takumi Kagawa found a positive correlation between HbEO and TT, TT/E2. Moderate EO exposure was associated with a decrease in E2 levels (52). Another study by Cao et al., which only explored the relationship between HbEO and TT, found that HbEO was positively correlated with TT, with the association being stronger in males than in females (53). In contrast, our study considered the distinct sex hormone levels across different sexes, age groups, and pubertal status, providing a more comprehensive exploration of the relationship between EO exposure and five sex hormones by sex-age and sex-puberty groups. Consistently, we observed that HbEO was significantly inversely associated with E2 and positively associated with TT/E2 and SHBG levels in adult males. Previous studies have pointed out that sperm production can be influenced by E2 (54, 55). Consequently, this analysis suggested EO exposure may affect sperm production in male adults consistent with previous animal experiments. For TT, our study did not find results consistent with the two aforementioned studies. This discrepancy is likely attributed to the fact that biologically available TT and E2 levels depend on SHBG concentrations. Therefore, in our regression models assessing the relationship between HbEO and TT or E2, we strictly controlled for SHBG. Our findings differ from previous studies, suggesting that the observed increase in TT levels among adult males exposed to HbEO may be mediated through SHBG. Further robust evidence is required to confirm these mechanisms. Additionally, our findings are consistent with results from animal studies, where plasma testosterone concentrations did not significantly change after EO exposure in rats (16). Notably, a prolonged estrus cycle and an increased percentage of the diestrus stage in the presence of EO exposure were observed in female mice (15, 56). Nonetheless, our research did not uncover any substantial correlation between EO exposure and sex hormones in female subjects. Previous papers have also shown different effects of other pollutants on male and female sex hormones (27, 57, 58).Such differences may occur due to the differences in hormonal regulatory networks exist significantly between males and females. In females, the high baseline levels of estrogen may help stabilize hormone dynamics by modulating the secretion of gonadotropic hormones such as luteinizing hormone (LH) and follicle-stimulating hormone (FSH), thereby mitigating the disruptive effects of EO (59). Additionally, estrogens may exert protective effects by activating their receptors (Estrogen Receptor (ER) α and ERβ), preserving the integrity of the endocrine system and reducing the oxidative stress or genotoxicity induced by EO exposure. Furthermore, EO may undergo distinct metabolic pathways in males and females. For example, variations in enzymatic activity due to gender differences could result in faster detoxification of EO in female systems. Moreover, lifestyle and environmental factors may play a role; women are more likely to use hormonal medications, such as oral contraceptives, which might modulate hormone levels and overshadow the effects of EO exposure. The dynamic fluctuations in hormone levels during specific life stages, such as premenopause and postmenopause, might also contribute to the difficulty in detecting EO’s effects through statistical analysis. Due to the limited research on the biological mechanisms of EO’s impact on sex hormones in experimental animals, it is challenging to elucidate the mechanistic approach behind the observed differences in EO’s effects on sex hormones between male and female populations. Such complexities underscore the need for further research to comprehensively understand the biological mechanisms underlying EO’s influence on sex hormones in both genders.

The biological processes through which EO influences endocrine hormone levels remain unclear. Endocrine disruptors can indirectly affect serum sex hormone levels through various mechanisms, such as regulating luteinizing hormone and follicle-stimulating hormone signaling, liver excretion processes, and the biofeedback processes of related hormones (60–64). Air pollutants, may primarily act on cholesterol metabolism pathways and hepatic function, thereby influencing sex hormone synthesis (65, 66). Specifically, long-term exposure to EO induces oxidative stress in the body, leading to increased hepatic lipid peroxidation and decreased glutathione reductase activity, which in turn affects lipid metabolism (67). Abnormal lipid metabolism can interfere with sex hormone synthesis. Additionally, EO exposure can trigger inflammation (68, 69). When inflammation occurs, pro-inflammatory cytokines typically activate the hypothalamic-pituitary-adrenal (HPA) axis. This activation helps prevent excessive inflammatory reactions by leveraging the anti-inflammatory effects of glucocorticoids. In this process, the HPA axis may also influence reproductive, growth, and thyroid functions, ultimately altering sex hormone levels. Conversely, abnormal sex hormone levels can modify inflammatory and immune responses (70). Future studies should explore the biological mechanisms involved in the relationship between EO and sex hormones in greater depth.

This study possesses multiple strengths. To the best of our understanding, this investigation represents the earliest utilization of the NHANES dataset to examine the correlation between the degree of EO exposure and sex hormones across a demographically comprehensive sample of the American population by sex-age or sex-puberty status. The database was meticulously filtered through stringent protocols, ensuring standard quality control measures. Large-scale databases, such as NHANES, have been instrumental to researchers and humanity. With their access to diverse populations and comprehensive datasets, these databases facilitate the identification of additional risk factors and disease biomarkers (71–76). Additionally, the levels of TT and E2 were assayed using ID-LC-MS/MS, offering enhanced precision compared to the immunoassay technique (21). Nevertheless, our study is subject to several constraints. Firstly, the lack of precise puberty status data within our study population hinders the thorough adjustment for the effects of puberty’s influence on this finding. In addition, this study carried out analyses using a derived “puberty status” classification, determined by the steroid hormone levels and menarcheal status. These criteria are not reliable for distinguishing pubertal from prepubertal status, as age and steroid hormone levels are not the most dependable indicators of pubertal progression (77). Consequently, unmeasured confounding variables associated with the unquantified puberty status persist. Secondly, as with other epidemiological analysis, our findings may be influenced by unmeasured confounding variables, such as job status and exposure to other chemical and physical pollutants. Thirdly, the absence of data on gonadotropins and key enzymes in steroidogenesis prevented us from investigating the fundamental biological mechanisms involved. Fourthly, The NHANES is a cross-sectional survey conducted by the Centers for Disease Control and Prevention (CDC) to estimate the health and nutritional status of the non-institutionalized population in the US, it is does not represent the situation in other countries or regions. Additionally, the NHANES data based on self-reported information such as BMI from individuals, potentially leading to recall bias. In the meanwhile, the measurement error of the exposure may have led our results biased towards the null.

## 5 Conclusions

HbEO was significantly inversely associated with E2 and positively associated with TT/E2 and SHBG levels in adult males. Such associations between HbEO with E2 and SHBG were non-linear in male adults. However, no significant correlation was detected between HbEO and sex steroids in different age female groups. Given the cross-sectional study design and the restricted sample size, additional research is required to validate and replicate these results. Furthermore, an in-depth exploration of the specific mechanisms by which EO affects sex hormones in male adults is essential. In addition, it is crucial for relevant U.S. agencies to strengthen adherence to EO exposure limits and regulatory frameworks, and to further refine corresponding research and rule-making for populations with prolonged exposure to EO.

## Data Availability

The original contributions presented in the study are included in the article/**Supplementary Material**. Further inquiries can be directed to the corresponding author.
