# Supplementary material for: Association between ethylene oxide exposure and serum sex hormone levels measured in a reference sample of the US general population
Source: Front Endocrinol (Lausanne). 2025 Apr 11;16:1533516. doi: 10.3389/fendo.2025.1533516 (PMC12023753; doi:10.3389/fendo.2025.1533516)
Supplement: Supplementary file 1 [file DataSheet1.docx]

Supplementary Material

# Supplementary Figures and Tables

##
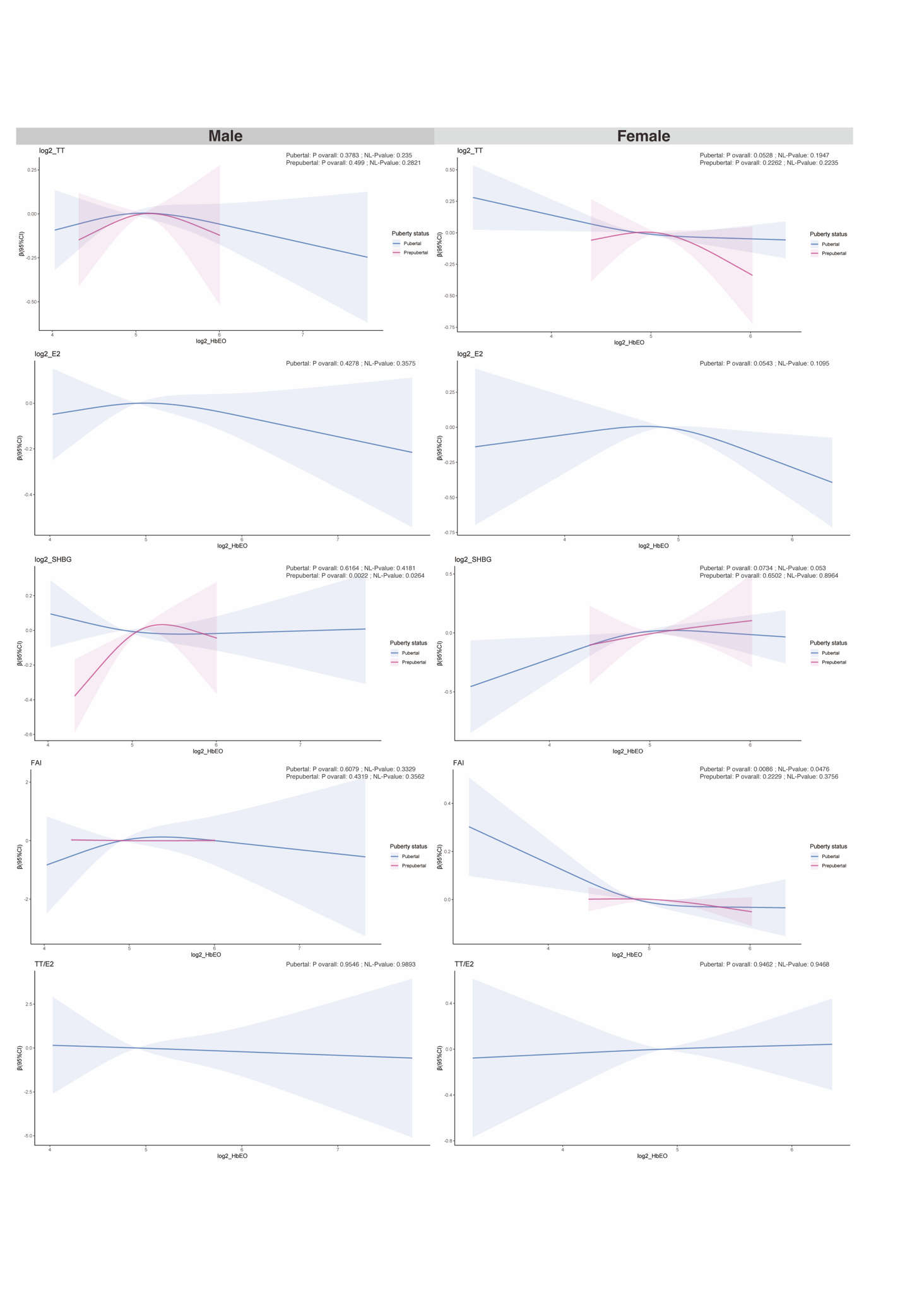
Supplementary Figures

**Supplementary Figure S1.** Analysis of the association between log2-HbEO and sex hormones by sex-puberty status in participants in NHANES 2013–2016 using the RCS. HbEO, Ethylene oxide hemoglobin adducts; TT, total testosterone; E2, total estradiol; SHBG, sex hormone binding globulin; FAI, free androgen index, was calculated as TT(ng /dL)/SHBG (nmol/L), TT/E2 was calculated as TT (ng /dL)/E2 (pm/ml).

## Supplementary Table

**Supplementary Table S1** Weighted quartiles of the log2-HbEO were calculated within each sex-age group and sex- puberty status.

| Group | Quartiles of log2-HbEO | Male | |  | Female | |
| --- | --- | --- | --- | --- | --- | --- |
|  |  | Range | N |  | Range | N |
| Children | Q1 | log2-HbEO≤4.75 | 57 |  | log2-HbEO≤4.75 | 64 |
|  | Q2 | 4.75<log2_HbEO≤5.02 | 60 |  | 4.75<log2-HbEO≤5.02 | 57 |
|  | Q3 | 5.02<log2-HbEO<5.33 | 60 |  | 5.02<log2-HbEO<5.34 | 57 |
|  | Q4 | log2-HbEO≥5.33 | 82 |  | log2-HbEO≥5.34 | 77 |
| Adolescents | Q1 | log2-HbEO≤4.52 | 64 |  | log2-HbEO≤4.53 | 82 |
|  | Q2 | 4.52<log2-HbEO≤4.89 | 86 |  | 4.53<log2-HbEO≤4.86 | 81 |
|  | Q3 | 4.89<log2-HbEO<5.42 | 88 |  | 4.86<log2-HbEO<5.21 | 76 |
|  | Q4 | log2-HbEO≥5.42 | 82 |  | log2-HbEO≥5.21 | 97 |
| Adults | Q1 | log2-HbEO≤4.50 | 310 |  | log2-HbEO≤4.41 | 299 |
|  | Q2 | 4.50<log2-HbEO≤4.98 | 346 |  | 4.41<log2-HbEO≤4.86 | 374 |
|  | Q3 | 4.98<log2-HbEO<6.07 | 445 |  | 4.86<log2-HbEO<5.58 | 427 |
|  | Q4 | log2-HbEO≥6.07 | 440 |  | log2-HbEO≥5.58 | 410 |
| Prepubertal | Q1 | log2-HbEO≤4.75 | 58 |  | log2-HbEO≤4.77 | 49 |
|  | Q2 | 4.75<log2-HbEO≤5.02 | 57 |  | 4.77<log2-HbEO≤5.02 | 45 |
|  | Q3 | 5.02<log2-HbEO<5.33 | 61 |  | 5.02<log2-HbEO<5.34 | 48 |
|  | Q4 | log2-HbEO≥5.33 | 82 |  | log2-HbEO≥5.34 | 63 |
| Pubertal | Q1 | log2-HbEO≤4.60 | 71 |  | log2-HbEO≤4.55 | 94 |
|  | Q2 | 4.60<log2-HbEO≤4.89 | 80 |  | 4.55<log2-HbEO≤4.86 | 92 |
|  | Q3 | 4.89<log2-HbEO<5.43 | 88 |  | 4.86<log2-HbEO<5.24 | 91 |
|  | Q4 | log2-HbEO≥5.43 | 82 |  | log2-HbEO≥5.24 | 109 |

Notes: Children (6–11 years), adolescents (12–19 years) and adult (>19 years). HbEO, ethylene oxide hemoglobin adducts; Q, Quartile

**Supplementary Table S2.** Detection frequencies of sex hormone indicators and HbEO in participants of 6-19 years old in NHANES 2013-2014 (%>LLOD)

| Items | LLOD | All  (n=4221) | Female | | |  | Male | | |  | Female | |  | Male | |
| --- | --- | --- | --- | --- | --- | --- | --- | --- | --- | --- | --- | --- | --- | --- | --- |
|  |  |  | Children  (n=255) | Adolescents  (n=336) | Adult  (n=1510) |  | Children  (n=259) | Adolescents  (n=320) | Adult  (n=1541) |  | Prepubertal  (n=205) | Pubertal  (n=386) |  | Prepubertal  (n=258) | Pubertal  (n=321) |
| TT | 0.75 ng/mL | 99.81(4213) | 99.61(254) | 100.0(336) | 99.87(1508) |  | 98.07(254) | 100.0(320) | 100(1541) |  | 99.51(204) | 100(386) |  | 98.06(253) | 100.0(321) |
| E_2_ | 2.994 pg/mL | 85.93(3627) | 51.76(132) | 99.11(333) | 86.56(1307) |  | 7.34(19) | 94.38(302) | 99.55(1534) |  | 40 (82) | 99.22(383) |  | 3.49(9) | 97.2(312) |
| SHBG | 0.800 nmol/L | 100(4221) | 100 | 100 | 100 |  | 100 | 100 | 100 |  | 100(205) | 100(386) |  | 100(258) | 100(321) |
| HbEO | 12.9 pmol/gHb | 97.49(4115) | 100(255) | 96.73(325) | 96.23(1453) |  | 99.23(257) | 99.38(318) | 97.79(1507) |  | 100(205) | 97.15(375) |  | 99.22(256) | 99.38(319) |

Notes: HbEO, ethylene oxide hemoglobin adducts; TT, total testosterone; E2, total estradiol; SHBG, sex hormone binding globulin; FAI, free androgen index, was calculated as TT(ng /dL)/SHBG (nmol/L), TT/E2 was calculated as TT (ng /dL)/E2 (pm/ml).

**Supplementary Table S3** Weighted sample characteristics by sex-puberty status in participants of 6-19 years old in NHANES 2013–2016 with serum sex hormones and HbEO.

| **Variable** | **All** | **Male** | | | |  | **Female** | | | |
| --- | --- | --- | --- | --- | --- | --- | --- | --- | --- | --- |
|  |  | **All** | **Prepubertal** | **Pubertal** | **P-value** |  | **All** | **Prepubertal** | **Pubertal** | **P-value** |
| **Age (yrs)** ^a^ | 12.76(0.19) | 12.68(0.20) | 8.75(0.14) | 15.23(0.14) | < 0.0001 |  | 12.83(0.25) | 8.32(0.16) | 14.92(0.19) | < 0.0001 |
| **BMI** ^a^ | 22.11(0.25) | 21.49(0.33) | 18.67(0.34) | 23.32(0.41) | < 0.0001 |  | 22.76(0.33) | 18.21(0.38) | 24.85(0.44) | < 0.0001 |
| **Race/ethnicity** ^b^ |  |  |  |  | 0.56 |  |  |  |  | 0.52 |
| White (Non-Hispanic) | 301(52.21) | 170(54.34) | 78(54.90) | 92(53.97) |  |  | 131(49.98) | 52(52.60) | 79(48.77) |  |
| Non-Hispanic Black | 245(12.28) | 126(12.20) | 49(10.76) | 77(13.14) |  |  | 119(12.36) | 41( 9.66) | 78(13.60) |  |
| Hispanics | 281(16.37) | 121(15.39) | 53(14.39) | 68(16.04) |  |  | 160(17.39) | 53(18.37) | 107(16.94) |  |
| Other | 343(19.15) | 162(18.07) | 78(19.95) | 84(16.85) |  |  | 181(20.27) | 59(19.37) | 122(20.69) |  |
| **PIR** ^a^ | 2.35(0.12) | 2.44(0.13) | 2.51(0.17) | 2.39(0.15) | 0.45 |  | 2.26(0.14) | 2.41(0.27) | 2.18(0.13) | 0.4 |
| **Serum cotinine (ng/mL)** ^a^ | 7.81(2.29) | 11.93(4.07) | 0.25(0.05) | 19.49(6.53) | 0.01 |  | 3.51(2.16) | 0.31(0.07) | 4.98(3.15) | 0.14 |
| **Cotinine exposure status** ^b^ |  |  |  |  | 0.04 |  |  |  |  | 0.67 |
| Exposed (> 0.015 ng/ml) | 756(64.67) | 404(69.9) | 169(63.05) | 235(73.88) |  |  | 352(59.56) | 126(57.52) | 226(60.07) |  |
| Unexposed (≤0.015 ng/ml) | 413(35.33) | 174(30.1) | 88(36.95) | 86(26.12) |  |  | 239(40.44) | 79(42.48) | 160(39.93) |  |
| **Six month time period** ^b^ |  |  |  |  | 0.53 |  |  |  |  | 0.83 |
| May 1 through October 31 | 281(48.53) | 281(48.53) | 136(55.55) | 145(50.98) |  |  | 314(53.13) | 105(57.67) | 209(59.08) |  |
| November 1 through April 30 | 298(51.47) | 298(51.47) | 122(44.45) | 176(49.02) |  |  | 277(46.87) | 100(42.33) | 177(40.92) |  |
| **Education level** ^b^ |  |  |  |  | < 0.0001 |  |  |  |  | < 0.0001 |
| High school or general educational development | 328(28.03) | 154(26.6) | 0(0.00) | 154(46.80) |  |  | 174(29.44) | 0( 0.00) | 174(50.32) |  |
| Less than high school | 810(69.23) | 406(70.12) | 258(100.00) | 148 (47.72) |  |  | 404(68.36) | 205(100.00) | 199( 47.64) |  |
| More than high school | 32(2.74) | 19(3.28) | 0(0.00) | 19(5.48) |  |  | 13(2.2) | 0(0.00) | 13(2.04) |  |
| **Energy intake** ^b^ |  |  |  |  | 0.08 |  |  |  |  | 0.09 |
| <2400 | 780(67.32) | 433(74.48) | 145(86.85) | 288(80.17) |  |  | 347(60.49) | 160(71.71) | 187(62.70) |  |
| ≥2400 | 269(23.85) | 98(16.28) | 26(13.15) | 72(19.83) |  |  | 171(31.07) | 56(28.29) | 115(37.30) |  |
| **Physical activity (METs-hour/week)** ^b^ |  |  |  |  | 0.01 |  |  |  |  | 0.66 |
| <200 | 24(1.50) | 14(1.89) | 2(30.26) | 12(3.14) |  |  | 10(1.12) | 1(3.25) | 9(1.95) |  |
| ≥200 | 543(52.81) | 266(51.59) | 4(69.74) | 262(96.86) |  |  | 277(53.98) | 11(96.75) | 266(98.05) |  |
| **Prescription medicine** ^b^ |  |  |  |  | 0.11 |  |  |  |  | 0.06 |
| No | 924(75.89) | 484(78.88) | 174(85.39) | 310(75.97) |  |  | 440(73.04) | 185(67.48) | 255(76.65) |  |
| Yes | 245(24.07) | 106(21.03) | 31(14.61) | 75(24.03) |  |  | 139(26.96) | 73(32.52) | 66(23.35) |  |
| **Serum sex hormones indices** ^b^ |  |  |  |  |  |  |  |  |  |  |
| TT((ng/dL) | 137.04(7.80) | 248.63(14.87) | 6.74(0.75) | 405.69(15.50) | < 0.0001 |  | 20.13(0.87) | 5.73(0.39) | 26.77(0.86) | < 0.0001 |
| SHBG (nmol/L) | 70.74(2.30) | 66.46(2.76) | 105.68(4.75) | 41.00(1.33) | < 0.0001 |  | 75.22(3.09) | 94.74(5.19) | 66.22(4.00) | < 0.001 |
| E2 (pg/mL) | 32.82(2.21) | 12.78(0.67) | 2.20(0.03) | 19.65(0.68) | < 0.0001 |  | 53.82(4.14) | 4.75(0.44) | 76.46(4.50) | < 0.0001 |
| FAI | 4.11(0.24) | 7.61(0.46) | 0.10(0.01) | 12.49(0.47) | < 0.0001 |  | 0.45(0.02) | 0.09(0.01) | 0.61(0.02) | < 0.0001 |
| TT/E2 | 8.37(0.34) | 15.32(0.66) | 3.07(0.35) | 23.28(0.77) | < 0.0001 |  | 1.08(0.11) | 1.65(0.17) | 0.82(0.14) | < 0.001 |
| **HbEO (pmol/gHb)** ^a^ | 42.44(2.95) | 46.54(4.35) | 34.99(1.08) | 54.03(7.06) | 0.01 |  | 38.15(3.59) | 36.08(1.33) | 39.11(5.22) | 0.58 |

^a^: Weighted mean value (±standard deviation [SD])

^b^: Frequencies (proportions) as appropriate

Abbreviations: PIR, the ratio of family income to poverty, was calculated by dividing family income by the poverty guidelines specific to family size, as well as the appropriate year and state. TT, total testosterone; E2, total estradiol; SHBG, sex hormone binding globulin; FAI, free androgen index, was calculated as total testosterone (ng /dL)/SHBG (nmol/L), TT/E2 was calculated as TT (ng /dL)/E2 (pm/ml). BMI, body mass index; HbEO, hemoglobin adducts of ethylene oxide;

**Supplementary Table S4.** Associations of continuous and quartiles of log2-HbEO with sex hormones in children and adolescents in NHANES 2013–2016.

| Sex hormones |  | Male | | | | |  | Female | | | | |
| --- | --- | --- | --- | --- | --- | --- | --- | --- | --- | --- | --- | --- |
|  |  | Children | |  | Adolescents | |  | Children | |  | Adolescents | |
|  |  | β(95%CI) | P-value |  | β(95%CI) | P-value |  | β(95%CI) | P-value |  | β(95%CI) | P-value |
| **TT** | Continuous log2-HbEO | 0.18(-0.13,0.49) | 0.23 |  | -0.04(-0.22, 0.14) | 0.62 |  | -0.27(-0.55, 0.02) | 0.06 |  | -0.05(-0.13,0.02) | 0.15 |
|  | Q1 | ref | ref |  | ref | ref |  | ref | ref |  | ref | ref |
|  | Q2 | -0.02(-0.88,0.84) | 0.96 |  | 0.36(-0.22, 0.94) | 0.20 |  | -0.2(-0.58,0.18) | 0.27 |  | 0.04(-0.29,0.38) | 0.80 |
|  | Q3 | -0.27(-0.96,0.43) | 0.41 |  | 0.13(-0.67, 0.93) | 0.73 |  | 0.06(-0.37,0.49) | 0.77 |  | -0.11(-0.35,0.13) | 0.34 |
|  | Q4 | -0.13(-0.70,0.45) | 0.63 |  | 0.23(-0.40, 0.85) | 0.45 |  | -0.3(-0.73,0.14) | 0.17 |  | -0.05(-0.26,0.16) | 0.64 |
|  | P for trend |  | 0.40 |  |  | 0.68 |  |  | 0.30 |  |  | 0.37 |
| **E2** | Continuous log2-HbEO | NA | NA |  | -0.05(-0.19, 0.09) | 0.46 |  | -0.29(-0.65, 0.07) | 0.10 |  | -0.12(-0.36, 0.11) | 0.27 |
|  | Q1 | NA | NA |  | ref | ref |  | ref | ref |  | ref | ref |
|  | Q2 | NA | NA |  | 0.07(-0.33, 0.47) | 0.72 |  | -0.15(-0.75, 0.46) | 0.60 |  | 0.4(-0.19, 0.99) | 0.16 |
|  | Q3 | NA | NA |  | 0.04(-0.43, 0.50) | 0.87 |  | -0.47(-0.94, 0.00) | **0.0497** |  | 0.17(-0.47, 0.82) | 0.57 |
|  | Q4 | NA | NA |  | 0.05(-0.38, 0.47) | 0.82 |  | -0.32(-0.76, 0.12) | 0.14 |  | 0.02(-0.56, 0.59) | 0.95 |
|  | P for trend |  | NA |  |  | 0.88 |  |  | 0.093 |  |  | 0.91 |
| **SHBG** | Continuous log2-HbEO | 0.08(-0.15, 0.31) | 0.46 |  | 0.01(-0.07, 0.10) | 0.72 |  | 0.06(-0.12, 0.24) | 0.47 |  | 0.05(-0.13, 0.24) | 0.53 |
|  | Q1 | ref | ref |  | ref | ref |  | ref | ref |  | ref | ref |
|  | Q2 | -0.06(-0.40, 0.28) | 0.71 |  | 0.09(-0.10, 0.28) | 0.34 |  | 0.24(-0.16, 0.65) | 0.22 |  | -0.02(-0.37, 0.33) | 0.90 |
|  | Q3 | -0.02(-0.33, 0.29) | 0.90 |  | -0.05(-0.25, 0.16) | 0.64 |  | 0.07(-0.20, 0.34) | 0.59 |  | 0.18(-0.40, 0.76) | 0.52 |
|  | Q4 | 0.11(-0.11, 0.33) | 0.30 |  | 0.07(-0.14, 0.29) | 0.48 |  | 0.08(-0.20, 0.36) | 0.53 |  | -0.07(-0.48, 0.35) | 0.73 |
|  | P for trend |  | 0.39 |  |  | 0.87 |  |  | 0.84 |  |  | 0.99 |
| **FAI** | Continuous log2-HbEO | 0.17(-0.17,0.51) | 0.30 |  | -0.31(-1.21, 0.60) | 0.48 |  | -0.04(-0.14,0.06) | 0.44 |  | -0.05(-0.11, 0.01) | 0.10 |
|  | Q1 | ref | ref |  | ref | ref |  | ref | ref |  | ref | ref |
|  | Q2 | -0.06(-0.69, 0.57) | 0.84 |  | 0.33(-1.56, 2.22) | 0.71 |  | -0.04(-0.17,0.09) | 0.54 |  | 0.03(-0.15, 0.20) | 0.74 |
|  | Q3 | -0.23(-0.69, 0.22) | 0.28 |  | 0.88(-1.59, 3.36) | 0.46 |  | -0.03(-0.12,0.06) | 0.52 |  | -0.14(-0.34, 0.05) | 0.14 |
|  | Q4 | -0.14(-0.50, 0.21) | 0.40 |  | 0.04(-2.49, 2.57) | 0.97 |  | -0.06(-0.17,0.06) | 0.30 |  | -0.03(-0.17, 0.11) | 0.69 |
|  | P for trend |  | 0.25 |  |  | 0.83 |  |  | 0.36 |  |  | 0.26 |
| **TT/E2** | Continuous log2-HbEO | NA | NA |  | -0.1( -1.62, 1.43) | 0.90 |  | 0(-0.28,0.29) | 0.98 |  | 0.06(-0.25,0.36) | 0.70 |
|  | Q1 | NA | NA |  | ref | ref |  | ref | ref |  | ref | ref |
|  | Q2 | NA | NA |  | 3.01( -0.93, 6.96) | 0.12 |  | -0.1(-0.90,0.70) | 0.79 |  | -0.7(-1.79,0.39) | 0.19 |
|  | Q3 | NA | NA |  | -0.31( -4.61, 4.00) | 0.88 |  | 0.79(-0.15,1.73) | 0.09 |  | -0.36(-1.75,1.02) | 0.58 |
|  | Q4 | NA | NA |  | 1.31( -2.60, 5.23) | 0.48 |  | -0.03(-0.53,0.48) | 0.91 |  | -0.38(-1.37,0.62) | 0.43 |
|  | P for trend |  | NA |  |  | 0.99 |  |  | 0.38 |  |  | 0.60 |

Notes: Children (6–11 years), adolescents (12–19 years). Estimates were presented as standardized coefficients and 95% confidence intervals (CIs) and were adjusted for age (continuous), race/ethnicity (categorical), education level (categorical), body mass index (BMI) category (categorical), poverty income ratio (PIR, continuous), cotinine (categorical), and the time of sample collection (categorical). “NA” indicates the estimates were not available in children as the detection frequency of E2 was < 50% in that group. Abbreviations: TT, total testosterone; E2, total estradiol; SHBG, sex hormone binding globulin; FAI, free androgen index, was calculated as total testosterone (ng /dL)/SHBG (nmol/L), TT/E2 was calculated as TT (ng /dL)/E2 (pm/ml). HbEO, hemoglobin adducts of ethylene oxide;

**Supplementary Table S5.** Associations of continuous and quartiles of log2-HbEO with sex hormones by sex-puberty status in participants of 6–19 years old in NHANES 2013–2016.

|  | | Male | | | | | | | | | |  | | Female | | | | | | | | | |
| --- | --- | --- | --- | --- | --- | --- | --- | --- | --- | --- | --- | --- | --- | --- | --- | --- | --- | --- | --- | --- | --- | --- | --- |
|  | | | Prepubertal | | | |  | Pubertal | | | | |  | Prepubertal | | | Prepubertal | | | | | | |
|  | | β(95%CI) | | P-value |  | | | | β(95%CI) | P-value | | |  | β(95%CI) | | P-value | |  | | β(95%CI) | P-value | | |
| TT | Continuous log2-HbEO | 0.04(-0.19,0.27) | | 0.71 |  | -0.06(-0.21, 0.09) | | | | | 0.42 | | |  | -0.14(-0.44,0.16) | 0.33 | |  | -0.05(-0.13,0.03) | | | 0.23 |  |
|  | Q1 | ref | | ref |  | ref | | | | | ref | | |  | ref | ref | |  | ref | | | ref |  |
|  | Q2 | 0.21(-0.47,0.88) | | 0.52 |  | 0.06(-0.26, 0.39) | | | | | 0.68 | | |  | -0.01(-0.42,0.40) | 0.96 | |  | -0.04(-0.36,0.28) | | | 0.80 |  |
|  | Q3 | 0.1(-0.34,0.54) | | 0.64 |  | 0.11(-0.29, 0.50) | | | | | 0.57 | | |  | 0.26(-0.18,0.69) | 0.21 | |  | -0.11(-0.30,0.09) | | | 0.27 |  |
|  | Q4 | 0.1(-0.42,0.62) | | 0.68 |  | -0.04(-0.45, 0.38) | | | | | 0.84 | | |  | -0.11(-0.56,0.35) | 0.60 | |  | -0.05(-0.25,0.14) | | | 0.58 |  |
|  | P for trend |  | | 0.72 |  |  | | | | | 0.94 | | |  |  | 0.94 | |  |  | | | 0.46 |  |
| E2 | Continuous log2-HbEO | NA | | NA |  | -0.06(-0.19, 0.07) | | | | | 0.34 | | |  | NA | NA | |  | -0.12(-0.35, 0.11) | | | 0.28 |  |
|  | Q1 | NA | | NA |  | ref | | | | | ref | | |  | NA | NA | |  | ref | | | ref |  |
|  | Q2 | NA | | NA |  | -0.01(-0.34, 0.33) | | | | | 0.97 | | |  | NA | NA | |  | 0.2(-0.37, 0.77) | | | 0.46 |  |
|  | Q3 | NA | | NA |  | 0.11(-0.20, 0.42) | | | | | 0.47 | | |  | NA | NA | |  | 0.1(-0.50, 0.69) | | | 0.73 |  |
|  | Q4 | NA | | NA |  | -0.06(-0.46, 0.33) | | | | | 0.73 | | |  | NA | NA | |  | -0.04(-0.60, 0.52) | | | 0.89 |  |
|  | P for trend |  | |  |  |  | | | | | 0.93 | | |  |  |  | |  |  | | | 0.86 |  |
| SHBG | Continuous log2-HbEO | 0.19(-0.01, 0.40) | | 0.06 |  | 0(-0.07, 0.08) | | | | | 0.91 | | |  | 0.04(-0.17, 0.25) | 0.66 | |  | 0.05(-0.11, 0.22) | | | 0.49 |  |
|  | Q1 | ref | | ref |  | ref | | | | | ref | | |  | ref | ref | |  | ref | | | ref |  |
|  | Q2 | 0.14(-0.13, 0.41) | | 0.28 |  | 0.01(-0.16, 0.18) | | | | | 0.89 | | |  | 0.2(-0.24, 0.64) | 0.33 | |  | -0.09(-0.36, 0.18) | | | 0.48 |  |
|  | Q3 | 0.08(-0.20, 0.36) | | 0.54 |  | -0.22(-0.42, -0.03) | | | | | 0.03 | | |  | 0.01(-0.28, 0.30) | 0.92 | |  | 0.17(-0.34, 0.68) | | | 0.48 |  |
|  | Q4 | 0.21(0.02, 0.40) | | 0.03 |  | 0.04(-0.16, 0.24) | | | | | 0.69 | | |  | 0.05(-0.26, 0.35) | 0.74 | |  | -0.1(-0.45, 0.24) | | | 0.54 |  |
|  | P for trend |  | | 0.079 |  |  | | | | | 0.59 | | |  |  | 0.90 | |  |  | | | 0.94 |  |
| FAI | Continuous log2-HbEO | -0.02(-0.07, 0.04) | | 0.55 |  | -0.29(-1.20, 0.62) | | | | | 0.51 | | |  | -0.02(-0.05,0.00) | 0.08 | |  | -0.04(-0.10, 0.02) | | | 0.14 |  |
|  | Q1 | ref | | ref |  | ref | | | | | ref | | |  | ref | ref | |  | ref | | | ref |  |
|  | Q2 | -0.04(-0.14, 0.05) | | 0.35 |  | 0.44(-1.30, 2.18) | | | | | 0.59 | | |  | 0.02(-0.04,0.07) | 0.58 | |  | 0.01(-0.15,0.17) | | | 0.91 |  |
|  | Q3 | -0.03(-0.08, 0.03) | | 0.29 |  | 1.66(-0.53, 3.86) | | | | | 0.13 | | |  | 0.01(-0.03,0.06) | 0.54 | |  | -0.12(-0.31,0.07) | | | 0.19 |  |
|  | Q4 | 0(-0.11, 0.10) | | 0.97 |  | -0.07(-2.48, 2.34) | | | | | 0.95 | | |  | -0.01(-0.05,0.02) | 0.40 | |  | -0.01(-0.14,0.12) | | | 0.87 |  |
|  | P for trend |  | | 0.97 |  |  | | | | | 0.72 | | |  |  | 0.44 | |  |  | | | 0.47 |  |
| TT/E2 | Continuous log2-HbEO | NA | | NA |  | 0.06( -1.43, 1.54) | | | | | 0.94 | | |  | NA | NA | |  | 0.06(-0.21,0.33) | | | 0.63 |  |
|  | Q1 | NA | | NA |  | ref | | | | | ref | | |  | NA | NA | |  | ref | | | ref |  |
|  | Q2 | NA | | NA |  | 0.91( -1.82, 3.64) | | | | | 0.48 | | |  | NA | NA | |  | -0.58(-1.49,0.34) | | | 0.20 |  |
|  | Q3 | NA | | NA |  | -1.31( -5.46, 2.83) | | | | | 0.50 | | |  | NA | NA | |  | -0.25(-1.46,0.95) | | | 0.66 |  |
|  | Q4 | NA | | NA |  | 0.41( -3.57, 4.39) | | | | | 0.83 | | |  | NA | NA | |  | -0.31(-1.17,0.56) | | | 0.46 |  |
|  | P for trend |  | |  |  |  | | | | | 0.852 | | |  |  |  | |  |  | | | 0.625 |  |

Notes: Puberty status was defined as “pubertal” if TT ≥ 50 ng/dL in males, E2 ≥ 20 pg/ml or menstrual period started in females, otherwise puberty status was defined as “prepubertal”. Estimates were presented as standardized coefficients and 95% confidence intervals (CIs) and were adjusted for age (continuous), race/ethnicity (categorical), education level (categorical), body mass index (BMI) category (categorical), poverty income ratio (PIR, continuous), cotinine (categorical), and the time of sample collection (categorical). Analyses for E2 were not performed in prepubertal participants as the detection frequency of E2 < 50% in that group. Q1, Q2, Q3 and Q4 represent 1st to 4th quartiles of log2-HbEO. “NA” indicates the estimates were not available in prepubertal participants as the detection frequency of E2 was < 50% in that group. Abbreviations: TT, total testosterone; E2, total estradiol; SHBG, sex hormone binding globulin; FAI, free androgen index, was calculated as TT(ng /dL)/SHBG (nmol/L), TT/E2 was calculated as TT (ng /dL)/E2 (pm/ml); HbEO, hemoglobin adducts of ethylene oxide;

**Supplementary Table S6.** Associations of continuous and quartiles of log2-HbEO with sex hormones by sex-age groups in participants in NHANES 2013–2016 after multiple imputation.

| **Sex hormones** |  | **Male** | | | | | | | |  | **Female** | | | | | | | |
| --- | --- | --- | --- | --- | --- | --- | --- | --- | --- | --- | --- | --- | --- | --- | --- | --- | --- | --- |
|  |  | **Children** | |  | **Adolescents** | |  | **Adults** | |  | **Children** | |  | **Adolescents** | |  | **Adults** | |
|  |  | **β(95%CI)** | **P-value** |  | **β(95%CI)** | **P-value** |  | **β(95%CI)** | **P-value** |  | **β(95%CI)** | **P-value** |  | **β(95%CI)** | **P-value** |  | **β(95%CI)** | **P-value** |
| **TT** | Continuous log2-HbEO | 0.28(-0.06,0.62) | 0.11 |  | 0.2(-0.03,0.43) | 0.09 |  | 0.29(-0.05,0.63) | 0.09 |  | 0.08(-0.13,0.28) | 0.45 |  | -0.01(-0.13, 0.10) | 0.81 |  | 0.02(-0.01, 0.04 | 0.22 |
|  | Q1 | ref | ref |  | ref | ref |  | ref | ref |  | ref | ref |  | ref | ref |  | ref | ref |
|  | Q2 | -0.26(-0.77,0.26) | 0.31 |  | -0.18(-0.60,0.24) | 0.38 |  | -0.01(-0.11, 0.10) | 0.92 |  | -0.16(-0.55,0.24) | 0.41 |  | 0.18(-0.08, 0.44) | 0.17 |  | -0.01(-0.11, 0.10) | 0.92 |
|  | Q3 | -0.31(-0.83,0.21) | 0.23 |  | -0.3(-0.70,0.11) | 0.14 |  | -0.02(-0.11, 0.06) | 0.53 |  | -0.31(-0.67,0.05) | 0.09 |  | 0.32( 0.04, 0.59) | **0.03** |  | -0.02(-0.11, 0.06) | 0.53 |
|  | Q4 | 0.07(-0.44,0.57) | 0.79 |  | 0.11(-0.42,0.64) | 0.67 |  | 0.12(-0.01, 0.25) | 0.07 |  | 0.02(-0.41,0.44) | 0.93 |  | 0.04(-0.21, 0.28) | 0.76 |  | 0.12(-0.01, 0.25) | 0.07 |
| **E2** | Continuous log2-HbEO | NA | NA |  | 0.01(-0.17,0.19) | 0.90 |  | 0.12( 0.07, 0.16) | **<0.0001** |  | -0.02(-0.19, 0.16) | 0.84 |  | 0.04(-0.08, 0.16) | 0.52 |  | -0.01(-0.07, 0.05) | 0.71 |
|  | Q1 | NA | NA |  | ref | ref |  | ref | ref |  | ref | ref |  | ref | ref |  | ref | ref |
|  | Q2 | NA | NA |  | -0.06(-0.47,0.35) | 0.76 |  | 0.07(-0.06, 0.19) | 0.28 |  | -0.02(-0.42, 0.37) | 0.91 |  | 0.27(-0.21, 0.75) | 0.26 |  | -0.15(-0.33, 0.02) | 0.08 |
|  | Q3 | NA | NA |  | -0.13(-0.56,0.29) | 0.52 |  | 0.17( 0.04, 0.30) | **0.01** |  | -0.12(-0.47, 0.23) | 0.48 |  | 0.16(-0.20, 0.52) | 0.36 |  | -0.1(-0.29, 0.09) | 0.28 |
|  | Q4 | NA | NA |  | 0(-0.46,0.46) | 1.00 |  | 0.46( 0.30, 0.62) | **<0.0001** |  | 0.06(-0.31, 0.44) | 0.72 |  | 0.06(-0.34, 0.46) | 0.76 |  | 0(-0.18, 0.18) | 0.97 |
| **SHBG** | Continuous log2-HbEO | 0.12(-0.11,0.35) | 0.30 |  | 0.13(-0.01,0.27) | 0.06 |  | 0.18( 0.05, 0.31) | **0.01** |  | 0.13(-0.07,0.32) | 0.21 |  | 0.12(-0.01, 0.24) | 0.07 |  | 0(-0.04, 0.04) | 0.94 |
|  | Q1 | ref | ref |  | ref | ref |  | ref | ref |  | ref | ref |  | ref | ref |  | ref | ref |
|  | Q2 | 0.26(-0.09,0.60) | 0.13 |  | -0.03(-0.12,0.06) | 0.53 |  | 0.27(-0.07, 0.62) | 0.11 |  | 0.25(-0.08,0.58) | 0.13 |  | 0.11(-0.14, 0.36) | 0.37 |  | -0.03(-0.12, 0.05) | 0.44 |
|  | Q3 | 0.14(-0.10,0.39) | 0.24 |  | -0.02(-0.14,0.10) | 0.73 |  | 0.17(-0.14, 0.48) | 0.26 |  | 0.13(-0.11,0.36) | 0.28 |  | 0.18(-0.11, 0.47) | 0.20 |  | -0.03(-0.14, 0.08) | 0.59 |
|  | Q4 | 0.1(-0.43,0.64) | 0.68 |  | 0(-0.09,0.09) | 0.98 |  | 0.2(-0.14, 0.54) | 0.23 |  | 0.13(-0.35,0.60) | 0.59 |  | 0.25(-0.02, 0.52) | 0.06 |  | 0.05(-0.09, 0.18) | 0.46 |
| **FAI** | Continuous log2-HbEO | 0.17(-0.21,0.55) | 0.37 |  | -0.01(-0.24, 0.22) | 0.93 |  | 0.29(-0.04,0.62) | 0.08 |  | -0.17(-0.49,0.14) | 0.26 |  | -0.11(-0.24, 0.03) | 0.12 |  | 0.02(-0.02, 0.05) | 0.37 |
|  | Q1 | ref | ref |  | ref | ref |  | ref | ref |  | ref | ref |  | ref | ref |  | ref | ref |
|  | Q2 | -0.5(-1.23,0.23) | 0.17 |  | 0.07(-0.40, 0.53) | 0.76 |  | -0.5(-1.23,0.23) | 0.17 |  | -0.28(-0.76,0.20) | 0.24 |  | 0.09(-0.29, 0.46) | 0.63 |  | 0.04(-0.07, 0.15) | 0.48 |
|  | Q3 | -0.44(-1.08,0.20) | 0.16 |  | 0.1(-0.27, 0.46) | 0.57 |  | -0.47(-1.09,0.15) | 0.14 |  | -0.45(-1.00,0.10) | 0.10 |  | 0.17(-0.20, 0.55) | 0.34 |  | -0.06(-0.19, 0.07) | 0.35 |
|  | Q4 | -0.03(-0.75,0.69) | 0.93 |  | 0.02(-0.43, 0.46) | 0.93 |  | 0.07(-0.62,0.76) | 0.85 |  | -0.35(-0.93,0.23) | 0.22 |  | -0.17(-0.49, 0.16) | 0.30 |  | 0.05(-0.08, 0.17) | 0.43 |
| **TT/E2** | Continuous log2-HbEO | NA | NA |  | 0.01(-0.01,0.03) | 0.39 |  | 0.05( 0.02, 0.08) | **<0.001** |  | 0.17(-0.06, 0.40) | 0.14 |  | 0(-0.18,0.17) | 0.95 |  | 0.03(-0.03, 0.09) | 0.37 |
|  | Q1 | NA | NA |  | ref | ref |  | ref | ref |  | ref | ref |  | ref | ref |  | ref | ref |
|  | Q2 | NA | NA |  | -0.06(-0.13,0.02) | 0.15 |  | 0.06(-0.07, 0.19) | 0.33 |  | -0.09(-0.51, 0.32) | 0.64 |  | -0.05(-0.49,0.39) | 0.82 |  | 0.16(-0.04, 0.37) | 0.11 |
|  | Q3 | NA | NA |  | -0.07(-0.15,0.02) | 0.10 |  | 0.07(-0.03, 0.17) | 0.14 |  | -0.14(-0.50, 0.22) | 0.41 |  | 0.23(-0.13,0.58) | 0.19 |  | 0.01(-0.17, 0.20) | 0.87 |
|  | Q4 | NA | NA |  | 0.02(-0.07,0.11) | 0.64 |  | 0.18( 0.07, 0.28) | **0.003** |  | 0.07(-0.37, 0.51) | 0.74 |  | 0.08(-0.35,0.50) | 0.70 |  | 0.09(-0.08, 0.27) | 0.29 |

Notes: Children (6–11 years), adolescents (12–19 years) and adult (>19 years). Estimates were presented as standardized coefficients and 95% confidence intervals (CIs) and were adjusted for age (continuous), race/ethnicity (categorical), education level (categorical), body mass index (BMI) category (categorical), poverty income ratio (PIR, continuous), cotinine (categorical), the time of sample collection (categorical). Q1, Q2, Q3 and Q4 represent 1st to 4th quartiles of log2-HbEO. Abbreviations: TT, total testosterone; E2, total estradiol; SHBG, sex hormone binding globulin; FAI, free androgen index, was calculated as total testosterone (ng /dL)/SHBG (nmol/L), TT/E2 was calculated as TT (ng /dL)/E2 (pm/ml); HbEO, hemoglobin adducts of ethylene oxide;

**Supplementary Table S7.** Associations of continuous and quartiles of log2-HbEO with sex hormones by sex-puberty status in participants of 6–19 years old in NHANES 2013–2016 after multiple imputation.

|  | | Male | | | | | | | | | |  | Female | | | | | | | | | | | |
| --- | --- | --- | --- | --- | --- | --- | --- | --- | --- | --- | --- | --- | --- | --- | --- | --- | --- | --- | --- | --- | --- | --- | --- | --- |
|  | | | Prepubertal | | | |  | Pubertal | | | | | |  | | Prepubertal | | Prepubertal | | | | | | |
|  | | β(95%CI) | | P-value |  | | | | β(95%CI) | P-value | | | |  | | β(95%CI) | P-value | |  | | β(95%CI) | P-value | | |
| TT | Continuous log2-HbEO | -0.05(-0.20,0.09) | | 0.46 |  | 0.08(-0.01, 0.17) | | | | | 0.09 | |  | | 0.02(-0.16,0.19) | | 0.86 | |  | 0.01(-0.12, 0.15) | | | 0.86 |  |
|  | Q1 | ref | | ref |  | ref | | | | | ref | |  | | ref | | ref | |  | ref | | | ref |  |
|  | Q2 | -0.04(-0.36,0.29) | | 0.82 |  | -0.07(-0.22, 0.08) | | | | | 0.35 | |  | | -0.15(-0.49,0.20) | | 0.37 | |  | 0.12(-0.15, 0.39) | | | 0.37 |  |
|  | Q3 | -0.21(-0.53,0.10) | | 0.17 |  | 0(-0.18, 0.18) | | | | | 0.98 | |  | | -0.19(-0.61,0.23) | | 0.36 | |  | 0.13(-0.11, 0.37) | | | 0.25 |  |
|  | Q4 | -0.07(-0.33,0.18) | | 0.53 |  | 0.08(-0.15, 0.31) | | | | | 0.48 | |  | | -0.11(-0.45,0.23) | | 0.50 | |  | 0.06(-0.23, 0.35) | | | 0.68 |  |
| E2 | Continuous log2-HbEO | NA | | NA |  | 0(-0.12,0.12) | | | | | 0.96 | |  | | NA | | NA | |  | -0.01(-0.15, 0.13) | | | 0.91 |  |
|  | Q1 | NA | | NA |  | ref | | | | | ref | |  | | NA | | NA | |  | ref | | | ref |  |
|  | Q2 | NA | | NA |  | 0.06(-0.20, 0.33) | | | | | 0.62 | |  | | NA | | NA | |  | 0.2(-0.19, 0.60) | | | 0.29 |  |
|  | Q3 | NA | | NA |  | -0.04(-0.26, 0.17) | | | | | 0.66 | |  | | NA | | NA | |  | 0.11(-0.22, 0.45) | | | 0.47 |  |
|  | Q4 | NA | | NA |  | 0(-0.19, 0.19) | | | | | 0.98 | |  | | NA | | NA | |  | 0.02(-0.33, 0.37) | | | 0.90 |  |
| SHBG | Continuous log2-HbEO | -0.12(-0.31, 0.07) | | 0.20 |  | 0.14(-0.06,0.33) | | | | | 0.17 | |  | | 0.14(-0.08, 0.35) | | 0.20 | |  | 0.02(-0.19,0.24) | | | 0.82 |  |
|  | Q1 | ref | | ref |  | ref | | | | | ref | |  | | ref | | ref | |  | ref | | | ref |  |
|  | Q2 | 0.25(-0.07, 0.58) | | 0.12 |  | 0.06(-0.25,0.36) | | | | | 0.71 | |  | | 0.07(-0.20, 0.33) | | 0.60 | |  | 0.3(-0.05,0.65) | | | 0.09 |  |
|  | Q3 | 0.12(-0.12, 0.37) | | 0.31 |  | -0.01(-0.36,0.33) | | | | | 0.94 | |  | | -0.09(-0.42, 0.24) | | 0.58 | |  | 0.14(-0.12,0.40) | | | 0.28 |  |
|  | Q4 | -0.19(-0.64, 0.26) | | 0.38 |  | 0.11(-0.24,0.45) | | | | | 0.54 | |  | | 0.08(-0.27, 0.43) | | 0.62 | |  | -0.15(-0.58,0.28) | | | 0.48 |  |
| FAI | Continuous log2-HbEO | 0.07(-0.22,0.37) | | 0.60 |  | -0.03(-0.29,0.23) | | | | | 0.81 | |  | | -0.12(-0.38,0.14) | | 0.34 | |  | -0.04(-0.18,0.10) | | | 0.57 |  |
|  | Q1 | ref | | ref |  | ref | | | | | ref | |  | | ref | | ref | |  | ref | | | ref |  |
|  | Q2 | -0.3(-0.84,0.24) | | 0.26 |  | -0.28(-0.71,0.15) | | | | | 0.20 | |  | | -0.21(-0.62,0.19) | | 0.28 | |  | 0.07(-0.28, 0.42) | | | 0.68 |  |
|  | Q3 | -0.34(-0.80,0.12) | | 0.14 |  | -0.22(-0.87,0.43) | | | | | 0.49 | |  | | -0.1(-0.64,0.44) | | 0.70 | |  | -0.04(-0.40, 0.33) | | | 0.83 |  |
|  | Q4 | 0.12(-0.41,0.66) | | 0.63 |  | -0.21(-0.67,0.25) | | | | | 0.36 | |  | | -0.19(-0.62,0.24) | | 0.36 | |  | -0.22(-0.58, 0.14) | | | 0.22 |  |
| TT/E2 | Continuous log2-HbEO | NA | | NA |  | 0.01(-0.05, 0.08 | | | | | 0.67 | |  | | NA | | NA | |  | 0.08(-0.08, 0.25) | | | 0.28 |  |
|  | Q1 | NA | | NA |  | ref | | | | | ref | |  | | NA | | NA | |  | ref | | | ref |  |
|  | Q2 | NA | | NA |  | -0.05(-0.20, 0.10) | | | | | 0.48 | |  | | NA | | NA | |  | -0.06(-0.38, 0.27) | | | 0.71 |  |
|  | Q3 | NA | | NA |  | -0.02(-0.12, 0.09) | | | | | 0.75 | |  | | NA | | NA | |  | 0.12(-0.21, 0.45) | | | 0.45 |  |
|  | Q4 | NA | | NA |  | -0.11(-0.29, 0.07) | | | | | 0.22 | |  | | NA | | NA | |  | 0.19(-0.08, 0.47) | | | 0.15 |  |

Notes: Puberty status was defined as “pubertal” if TT ≥ 50 ng/dL in males, E2 ≥ 20 pg/ml or menstrual period started in females, otherwise puberty status was defined as “prepubertal”. Estimates were presented as standardized coefficients and 95% confidence intervals (CIs) and were adjusted for age (continuous), race/ethnicity (categorical), education level (categorical), body mass index (BMI) category (categorical), poverty income ratio (PIR, continuous), cotinine (categorical), and the time of sample collection (categorical). Analyses for E2 were not performed in prepubertal participants as the detection frequency of E2 < 50% in that group. Q1, Q2, Q3 and Q4 represent 1st to 4th quartiles of log2-HbEO. “NA” indicates the estimates were not available in prepubertal participants as the detection frequency of E2 was < 50% in that group. Abbreviations: TT, total testosterone; E2, total estradiol; SHBG, sex hormone binding globulin; FAI, free androgen index, was calculated as TT (ng /dL)/SHBG (nmol/L), TT/E2 was calculated as TT (ng /dL)/E2 (pm/ml); HbEO, hemoglobin adducts of ethylene oxide;

**Supplementary Table S8.** Associations of continuous and quartiles of log2-HbEO with sex hormones by sex-age groups in participants in NHANES 2013–2016  adjusted energy intake, physical activity, and prescription medication additionally.

| **Sex hormones** |  | **Male** | | | | | | | |  | **Female** | | | | | | | |
| --- | --- | --- | --- | --- | --- | --- | --- | --- | --- | --- | --- | --- | --- | --- | --- | --- | --- | --- |
|  |  | **Children** | |  | **Adolescents** | |  | **Adults** | |  | **Children** | |  | **Adolescents** | |  | **Adults** | |
|  |  | **β(95%CI)** | **P-value** |  | **β(95%CI)** | **P-value** |  | **β(95%CI)** | **P-value** |  | **β(95%CI)** | **P-value** |  | **β(95%CI)** | **P-value** |  | **β(95%CI)** | **P-value** |
| **TT** | Continuous log2-HbEO | 0.17(-0.17, 0.50) | 0.31 |  | 0.02(-0.27, 0.31) | 0.88 |  | 0.01(-0.03, 0.04) | 0.66 |  | -0.2(-0.44, 0.03) | 0.08 |  | -0.1(-0.22,0.02) | 0.09 |  | -0.064(-0.13, 0.001) | 0.052 |
|  | Q1 | ref | ref |  | ref | ref |  | ref | ref |  | ref | ref |  | ref | ref |  | ref | ref |
|  | Q2 | 0.55(-0.10, 1.21) | 0.09 |  | 0.5(-0.21, 1.21) | 0.15 |  | -0.01(-0.13, 0.11) | 0.87 |  | -0.13(-0.44, 0.17) | 0.34 |  | -0.02(-0.40,0.37) | 0.93 |  | -0.13(-0.33, 0.07) | 0.20 |
|  | Q3 | 0(-0.49, 0.49) | 1.00 |  | 0.29(-0.50, 1.08) | 0.44 |  | 0.02(-0.07, 0.11) | 0.66 |  | 0.05(-0.40, 0.49) | 0.82 |  | -0.1(-0.40,0.20) | 0.47 |  | -0.26(-0.48, -0.05) | 0.02 |
|  | Q4 | 0.14(-0.33, 0.61) | 0.52 |  | 0.3(-0.50, 1.09) | 0.43 |  | -0.02(-0.15, 0.10) | 0.70 |  | -0.12(-0.44, 0.20) | 0.42 |  | -0.14(-0.39,0.12) | 0.26 |  | -0.19(-0.39, 0.00) | 0.051 |
| **E2** | Continuous log2-HbEO | NA | NA |  | -0.04(-0.20, 0.13) | 0.64 |  | -0.04(-0.07, -0.01) | 0.02 |  | -0.21(-0.55, 0.12) | 0.19 |  | -0.22(-0.46, 0.03) | 0.08 |  | 0.03(-0.16, 0.21) | 0.74 |
|  | Q1 | NA | NA |  | ref | ref |  | ref | **ref** |  | ref | ref |  | ref | ref |  | ref | ref |
|  | Q2 | NA | NA |  | 0.25(-0.17, 0.68) | 0.21 |  | -0.15(-0.26, -0.05) | 0.01 |  | -0.31(-0.93, 0.32) | 0.30 |  | 0.53(-0.11, 1.18) | 0.10 |  | -0.16(-0.69, 0.36) | 0.51 |
|  | Q3 | NA | NA |  | 0.17(-0.28, 0.61) | 0.42 |  | -0.16(-0.29, -0.03) | 0.02 |  | -0.58(-1.13, -0.03) | 0.04 |  | 0(-0.71, 0.72) | 0.99 |  | -0.56(-1.16, 0.04) | 0.06 |
|  | Q4 | NA | NA |  | 0.14(-0.36, 0.63) | 0.56 |  | -0.19(-0.32, -0.06) | **0.01** |  | -0.2(-0.68, 0.29) | 0.38 |  | -0.04(-0.63, 0.55) | 0.88 |  | 0.07(-0.50, 0.63) | 0.81 |
| **SHBG** | Continuous log2-HbEO | 0.17(-0.12, 0.46) | 0.23 |  | -0.03(-0.17, 0.11) | 0.68 |  | 0.08(0.05,0.12) | **<0.001** |  | 0.22(-0.02, 0.46) | 0.07 |  | 0.15(-0.13, 0.44) | 0.27 |  | 0.02(-0.06,0.10) | 0.59 |
|  | Q1 | ref | ref |  | ref | ref |  | ref | **ref** |  | ref | ref |  | ref | ref |  | ref | ref |
|  | Q2 | -0.18(-0.65, 0.28) | 0.41 |  | -0.02(-0.32, 0.28) | 0.90 |  | 0.01(-0.12,0.13) | **0.93** |  | 0.47(0.01, 0.93) | 0.046 |  | -0.07(-0.59, 0.45) | 0.77 |  | -0.12(-0.42,0.18) | 0.40 |
|  | Q3 | -0.04(-0.47, 0.40) | 0.86 |  | -0.1(-0.36, 0.16) | 0.42 |  | -0.05(-0.22,0.12) | 0.51 |  | 0.24(-0.15, 0.62) | 0.20 |  | 0.42(-0.34, 1.17) | 0.25 |  | 0.12(-0.16,0.39) | 0.38 |
|  | Q4 | 0.06(-0.23, 0.34) | 0.68 |  | 0(-0.25, 0.26) | 0.98 |  | 0.26(0.12,0.41) | 0.001 |  | 0.27(-0.08, 0.63) | 0.12 |  | -0.01(-0.50, 0.49) | 0.97 |  | 0.1(-0.24,0.44) | 0.54 |
| **FAI** | Continuous log2-HbEO | -0.03(-0.51, 0.45) | 0.89 |  | 0.04(-0.29, 0.38) | 0.78 |  | -0.03(-0.06, 0.01) | 0.13 |  | -0.47(-0.87, -0.07) | **0.02** |  | -0.23(-0.50, 0.04) | 0.09 |  | -0.08(-0.18, 0.01) | 0.09 |
|  | Q1 | ref | ref |  | ref | ref |  | ref | ref |  | ref | ref |  | ref | ref |  | ref | ref |
|  | Q2 | 0.76(-0.16, 1.67) | 0.10 |  | 0.52(-0.23, 1.26) | 0.16 |  | -0.01(-0.12, 0.10) | 0.83 |  | -0.7(-1.25, -0.15) | 0.02 |  | 0.04(-0.50, 0.59) | 0.86 |  | -0.03(-0.24, 0.19) | 0.78 |
|  | Q3 | 0.04(-0.64, 0.72) | 0.90 |  | 0.38(-0.39, 1.15) | 0.30 |  | 0.04(-0.07, 0.15) | 0.44 |  | -0.24(-0.81, 0.33) | 0.38 |  | -0.46(-1.21, 0.28) | 0.20 |  | -0.36(-0.62, -0.10) | 0.01 |
|  | Q4 | 0.08(-0.56, 0.72) | 0.80 |  | 0.29(-0.56, 1.14) | 0.47 |  | -0.13(-0.27, 0.00) | 0.05 |  | -0.45(-1.01, 0.11) | 0.11 |  | -0.13(-0.64, 0.38) | 0.58 |  | -0.27(-0.57, 0.03) | 0.07 |
| **TT/E2** | Continuous log2-HbEO | NA | NA |  | 0.04(-0.12, 0.20) | 0.58 |  | 0.09(0.05, 0.12) | <0.0001 |  | 0.01(-0.26, 0.28) | 0.94 |  | 0.15(-0.15,0.45) | 0.31 |  | -0.09(-0.26,0.08) | 0.29 |
|  | Q1 | NA | NA |  | ref | ref |  | ref | ref |  | ref | ref |  | ref | ref |  | ref | ref |
|  | Q2 | NA | NA |  | 0.24(-0.23, 0.70) | 0.29 |  | 0.15(-0.02,0.32) | 0.08 |  | 0.17(-0.39, 0.73) | 0.51 |  | -0.56(-1.17,0.04) | 0.06 |  | 0.02(-0.52,0.55) | 0.95 |
|  | Q3 | NA | NA |  | 0.08(-0.38, 0.54) | 0.72 |  | 0.15(0.02,0.29) | 0.03 |  | 0.62(0.15, 1.10) | 0.02 |  | -0.02(-0.70,0.66) | 0.95 |  | 0.32(-0.18,0.81) | 0.19 |
|  | Q4 | NA | NA |  | 0.16(-0.25, 0.57) | 0.41 |  | 0.29(0.13,0.46) | **0.002** |  | 0.08(-0.34, 0.49) | 0.69 |  | -0.1(-0.68,0.48) | 0.71 |  | -0.24(-0.79,0.30) | 0.36 |

Notes: Children (6–11 years), adolescents (12–19 years) and adult (>19 years). Estimates were presented as standardized coefficients and 95% confidence intervals (CIs) and were adjusted for age (continuous), race/ethnicity (categorical), education level (categorical), body mass index (BMI) category (categorical), poverty income ratio (PIR, continuous), cotinine (categorical), the time of sample collection (categorical), energy intake (categorical), physical activity (categorical), and prescription medication (categorical). Q1, Q2, Q3 and Q4 represent 1st to 4th quartiles of log2-HbEO. Abbreviations: TT, total testosterone; E2, total estradiol; SHBG, sex hormone binding globulin; FAI, free androgen index, was calculated as TT (ng /dL)/SHBG (nmol/L), TT/E2 was calculated as TT (ng /dL)/E2 (pm/ml); HbEO, hemoglobin adducts of ethylene oxide;

**Supplementary Table S9.** Associations of continuous and quartiles of log2-HbEO with sex hormones by sex-puberty status in participants in NHANES 2013–2016  adjusted energy intake (categorical), physical activity (categorical), and prescription medication (categorical) additionally.

|  | | Male | | | | | | | | | |  | | Female | | | | | | | | | |
| --- | --- | --- | --- | --- | --- | --- | --- | --- | --- | --- | --- | --- | --- | --- | --- | --- | --- | --- | --- | --- | --- | --- | --- |
|  | | | Prepubertal | | | |  | Pubertal | | | | |  | Prepubertal | | | Prepubertal | | | | | | |
|  | | β(95%CI) | | P-value |  | | | | β(95%CI) | P-value | | |  | β(95%CI) | | P-value | |  | | β(95%CI) | P-value | | |
| TT | Continuous log2-HbEO | 0.01(-0.22,0.24) | | 0.91 |  | -0.06(-0.22, 0.10) | | | | | 0.43 | | |  | -0.08(-0.34,0.18) | 0.50 | |  | -0.09(-0.20,0.03) | | | 0.14 |  |
|  | Q1 | ref | | ref |  | ref | | | | | ref | | |  | ref | ref | |  | ref | | | ref |  |
|  | Q2 | 0.33(-0.30,0.97) | | 0.27 |  | 0.01(-0.30, 0.31) | | | | | 0.96 | | |  | -0.01(-0.38,0.36) | 0.96 | |  | -0.05(-0.44,0.34) | | | 0.78 |  |
|  | Q3 | 0.07(-0.36,0.49) | | 0.74 |  | 0.08(-0.26, 0.42) | | | | | 0.62 | | |  | 0.26(-0.24,0.77) | 0.26 | |  | -0.06(-0.32,0.21) | | | 0.64 |  |
|  | Q4 | 0.18(-0.37,0.72) | | 0.49 |  | -0.05(-0.44, 0.35) | | | | | 0.80 | | |  | 0.05(-0.38,0.49) | 0.79 | |  | -0.12(-0.35,0.10) | | | 0.26 |  |
| E2 | Continuous log2-HbEO | NA | | NA |  | -0.06(-0.18, 0.06) | | | | | 0.29 | | |  | NA | NA | |  | -0.19(-0.44,0.07) | | | 0.13 |  |
|  | Q1 | NA | | NA |  | ref | | | | | ref | | |  | NA | NA | |  | ref | | | ref |  |
|  | Q2 | NA | | NA |  | 0.01(-0.32, 0.34) | | | | | 0.94 | | |  | NA | NA | |  | 0.47(-0.20, 1.14) | | | 0.15 |  |
|  | Q3 | NA | | NA |  | 0.09(-0.15, 0.34) | | | | | 0.41 | | |  | NA | NA | |  | 0.1(-0.62, 0.82) | | | 0.77 |  |
|  | Q4 | NA | | NA |  | -0.02(-0.38, 0.35) | | | | | 0.93 | | |  | NA | NA | |  | -0.09(-0.68, 0.50) | | | 0.74 |  |
| SHBG | Continuous log2-HbEO | 0.24(-0.05, 0.52) | | 0.10 |  | -0.05(-0.17, 0.08) | | | | | 0.43 | | |  | 0.2(-0.09, 0.48) | 0.15 | |  | 0.16(-0.12, 0.45) | | | 0.24 |  |
|  | Q1 | ref | | ref |  | ref | | | | | ref | | |  | ref | ref | |  | ref | | | ref |  |
|  | Q2 | -0.01(-0.41, 0.38) | | 0.95 |  | -0.06(-0.34, 0.21) | | | | | 0.64 | | |  | 0.389(-0.212, 0.990) | 0.174 | |  | -0.22(-0.66, 0.21) | | | 0.28 |  |
|  | Q3 | 0.08(-0.28, 0.44) | | 0.65 |  | -0.21(-0.47, 0.05) | | | | | 0.10 | | |  | 0.177(-0.250, 0.604) | 0.366 | |  | 0.42(-0.24, 1.08) | | | 0.19 |  |
|  | Q4 | 0.14(-0.10, 0.37) | | 0.23 |  | -0.05(-0.28, 0.17) | | | | | 0.62 | | |  | 0.212(-0.225, 0.649) | 0.296 | |  | -0.09(-0.54, 0.36) | | | 0.67 |  |
| FAI | Continuous log2-HbEO | -0.22(-0.64,0.20) | | 0.27 |  | -0.02(-0.24, 0.20) | | | | | 0.84 | | |  | -0.28(-0.68,0.13) | 0.16 | |  | -0.23(-0.49, 0.04) | | | 0.09 |  |
|  | Q1 | ref | | ref |  | ref | | | | | ref | | |  | ref | ref | |  | ref | | | ref |  |
|  | Q2 | 0.35(-0.54,1.24) | | 0.41 |  | 0.06(-0.34, 0.45) | | | | | 0.76 | | |  | -0.4(-1.17,0.37) | 0.27 | |  | 0.15(-0.36,0.66) | | | 0.54 |  |
|  | Q3 | -0.01(-0.61,0.58) | | 0.96 |  | 0.25(-0.13, 0.64) | | | | | 0.18 | | |  | 0.09(-0.49,0.66) | 0.74 | |  | -0.43(-1.11,0.25) | | | 0.19 |  |
|  | Q4 | 0.04(-0.64,0.73) | | 0.90 |  | 0(-0.43, 0.43) | | | | | 0.99 | | |  | -0.16(-0.84,0.52) | 0.60 | |  | -0.04(-0.48,0.40) | | | 0.83 |  |
| TT/E2 | Continuous log2-HbEO | NA | | NA |  | -0.03(-0.12, 0.07) | | | | | 0.59 | | |  | NA | NA | |  | 0.14(-0.17,0.44) | | | 0.35 |  |
|  | Q1 | NA | | NA |  | ref | | | | | ref | | |  | NA | NA | |  | ref | | | ref |  |
|  | Q2 | NA | | NA |  | -0.04(-0.32, 0.25) | | | | | 0.79 | | |  | NA | NA | |  | -0.57(-1.17,0.03) | | | 0.06 |  |
|  | Q3 | NA | | NA |  | -0.12(-0.45, 0.21) | | | | | 0.44 | | |  | NA | NA | |  | -0.07(-0.74,0.61) | | | 0.84 |  |
|  | Q4 | NA | | NA |  | -0.06(-0.31, 0.19) | | | | | 0.63 | | |  | NA | NA | |  | -0.05(-0.64,0.54) | | | 0.85 |  |

Notes: Puberty status was defined as “pubertal” if TT ≥ 50 ng/dL in males, E2 ≥ 20 pg/ml or menstrual period started in females, otherwise puberty status was defined as “prepubertal”. Estimates were presented as standardized coefficients and 95% confidence intervals (CIs) and were adjusted for age (continuous), race/ethnicity (categorical), education level (categorical), body mass index (BMI) category (categorical), poverty income ratio (PIR, continuous), cotinine (categorical), and the time of sample collection (categorical). Analyses for E2 were not performed in prepubertal participants as the detection frequency of E2 < 50% in that group. Q1, Q2, Q3 and Q4 represent 1st to 4th quartiles of log2-HbEO. “NA” indicates the estimates were not available in prepubertal participants as the detection frequency of E2 was < 50% in that group. Abbreviations: TT, total testosterone; E2, total estradiol; SHBG, sex hormone binding globulin; FAI, free androgen index, was calculated as TT (ng /dL)/SHBG (nmol/L), TT/E2 was calculated as TT (ng /dL)/E2 (pm/ml); HbEO, hemoglobin adducts of ethylene oxide;

**Supplementary Table S10.** Associations of continuous and quartiles of log2-HbEO with sex hormones by sex-age groups in NHANES 2013–2016 adjusted exposure of bisphenol A, phthalates and polychlorinated biphenyls additionally.

| **Sex hormones** |  | **Male** | | | | | | | |  | **Female** | | | | | | | |
| --- | --- | --- | --- | --- | --- | --- | --- | --- | --- | --- | --- | --- | --- | --- | --- | --- | --- | --- |
|  |  | **Children** | |  | **Adolescents** | |  | **Adults** | |  | **Children** | |  | **Adolescents** | |  | **Adults** | |
|  |  | **β(95%CI)** | **P-value** |  | **β(95%CI)** | **P-value** |  | **β(95%CI)** | **P-value** |  | **β(95%CI)** | **P-value** |  | **β(95%CI)** | **P-value** |  | **β(95%CI)** | **P-value** |
| **TT** | Continuous log2-HbEO | -0.15(-0.64, 0.34) | 0.53 |  | 0.26(0.04, 0.48) | **0.03** |  | 0.03(-0.01,0.06) | 0.15 |  | 0.05(-0.25,0.35) | 0.74 |  | 0.02(-0.14, 0.18) | 0.76 |  | 0.06( 0.01, 0.12) | **0.03** |
|  | Q1 | ref | ref |  | ref | ref |  | ref | ref |  | ref | ref |  | ref | ref |  | ref | ref |
|  | Q2 | -0.54(-1.23,0.15) | 0.12 |  | 0.11(-0.34, 0.57) | 0.56 |  | 0.03(-0.14,0.19) | 0.73 |  | -0.17(-0.66,0.31) | 0.45 |  | -0.08(-0.41, 0.24) | 0.54 |  | -0.02(-0.15, 0.11) | 0.71 |
|  | Q3 | -0.37(-1.02,0.28) | 0.24 |  | 0.14(-0.32, 0.59) | 0.50 |  | -0.04(-0.17,0.09) | 0.48 |  | -0.09(-0.61,0.42) | 0.71 |  | 0(-0.36, 0.35) | 0.99 |  | 0.01(-0.16, 0.18) | 0.92 |
|  | Q4 | -0.46(-1.26,0.34) | 0.24 |  | 0.38(-0.13, 0.90) | 0.12 |  | 0.15(0.00,0.30) | 0.05 |  | 0.02(-0.54,0.58) | 0.93 |  | -0.02(-0.38, 0.35) | 0.92 |  | 0.18(-0.01, 0.36) | 0.06 |
| **E2** | Continuous log2-HbEO | NA | NA |  | 0.17(-0.04,0.39) | 0.10 |  | 0.06(0.03, 0.10) | **0.004** |  | 0.03(-0.23, 0.30) | 0.79 |  | 0.17(-0.11, 0.44) | 0.19 |  | -0.01(-0.13, 0.11) | 0.84 |
|  | Q1 | NA | NA |  | ref | ref |  | ref | ref |  | ref | ref |  | ref | ref |  | ref | ref |
|  | Q2 | NA | NA |  | 0.14(-0.23,0.50) | 0.39 |  | -0.05(-0.18, 0.07) | 0.37 |  | -0.33(-0.95, 0.30) | 0.28 |  | -0.01(-0.76, 0.74) | 0.97 |  | 0.07(-0.35, 0.49) | 0.70 |
|  | Q3 | NA | NA |  | -0.07(-0.39,0.26) | 0.62 |  | 0.02(-0.10, 0.15) | 0.67 |  | 0(-0.53, 0.53) | 0.99 |  | 0.22(-0.53, 0.97) | 0.49 |  | 0.15(-0.15, 0.44) | 0.28 |
|  | Q4 | NA | NA |  | 0.55(-0.11,1.22) | 0.09 |  | 0.21( 0.06, 0.36) | 0.01 |  | -0.03(-0.52, 0.46) | 0.90 |  | 0.24(-0.54, 1.01) | 0.47 |  | 0.04(-0.41, 0.49) | 0.86 |
| **SHBG** | Continuous log2-HbEO | -0.12(-0.45, 0.20) | 0.43 |  | 0.24( 0.01, 0.46) | **0.04** |  | 0.05( 0.02, 0.08) | **0.01** |  | 0.1(-0.14, 0.34) | 0.40 |  | -0.14(-0.28, 0.00) | 0.06 |  | -0.03(-0.10, 0.03) | 0.25 |
|  | Q1 | ref | ref |  | ref | ref |  | ref | ref |  | ref | ref |  | ref | ref |  | ref | ref |
|  | Q2 | 0.41( 0.02, 0.79) | 0.04 |  | 0(-0.38, 0.39) | 0.98 |  | -0.07(-0.25, 0.11) | 0.39 |  | -0.35(-0.83, 0.12) | 0.13 |  | -0.08(-0.47, 0.32) | 0.64 |  | 0.03(-0.13, 0.19) | 0.71 |
|  | Q3 | 0.15(-0.15, 0.46) | 0.29 |  | 0.13(-0.21, 0.47) | 0.40 |  | 0.09(-0.03, 0.20) | 0.11 |  | -0.25(-0.65, 0.16) | 0.21 |  | -0.1(-0.42, 0.23) | 0.50 |  | -0.04(-0.23, 0.15) | 0.65 |
|  | Q4 | -0.23(-0.80, 0.33) | 0.39 |  | 0.43(-0.10, 0.95) | 0.09 |  | 0.11(-0.05, 0.26) | 0.15 |  | 0.2(-0.25, 0.65) | 0.37 |  | -0.26(-0.71, 0.19) | 0.21 |  | 0.07(-0.16, 0.30) | 0.51 |
| **FAI** | Continuous log2-HbEO | -0.02(-0.76, 0.72) | 0.96 |  | 0.02(-0.28,0.33) | 0.87 |  | -0.02(-0.11, 0.06) | 0.56 |  | -0.05(-0.34,0.25) | 0.75 |  | 0.15(-0.05, 0.35) | 0.12 |  | 0.09( 0.01, 0.16) | **0.03** |
|  | Q1 | ref | ref |  | ref | ref |  | ref | ref |  | ref | ref |  | ref | ref |  | ref | ref |
|  | Q2 | -0.96(-1.82,-0.11) | 0.03 |  | 0.11(-0.44,0.66) | 0.65 |  | -0.13(-0.36, 0.10) | 0.23 |  | 0.17(-0.41,0.74) | 0.55 |  | -0.01(-0.43, 0.41) | 0.95 |  | -0.04(-0.23, 0.15) | 0.62 |
|  | Q3 | -0.53(-1.30, 0.25) | 0.17 |  | 0.01(-0.54,0.56) | 0.96 |  | -0.16(-0.37, 0.05) | 0.11 |  | 0.14(-0.44,0.73) | 0.61 |  | 0.09(-0.30, 0.49) | 0.59 |  | 0.04(-0.18, 0.25) | 0.70 |
|  | Q4 | -0.22(-1.33, 0.90) | 0.69 |  | -0.03(-0.62,0.57) | 0.92 |  | -0.14(-0.45, 0.17) | 0.32 |  | -0.17(-0.73,0.40) | 0.54 |  | 0.22(-0.36, 0.80) | 0.40 |  | 0.12(-0.13, 0.38) | 0.30 |
| **TT/E2** | Continuous log2-HbEO | NA | NA |  | 0.1(-0.09, 0.30) | 0.27 |  | 0.16(0.02,0.30) | **0.03** |  | 0.04(-0.30, 0.38) | 0.80 |  | -0.15(-0.36,0.06) | 0.14 |  | 0.07(-0.06, 0.20) | 0.25 |
|  | Q1 | NA | NA |  | ref | ref |  | ref | ref |  | ref | ref |  | ref | ref |  | ref | ref |
|  | Q2 | NA | NA |  | -0.02(-0.41, 0.37) | 0.91 |  | 0.33(-0.05,0.72) | 0.08 |  | 0.05(-0.58, 0.69) | 0.86 |  | -0.07(-0.64,0.50) | 0.77 |  | -0.09(-0.48, 0.30) | 0.61 |
|  | Q3 | NA | NA |  | 0.22(-0.14, 0.57) | 0.19 |  | -0.02(-0.42,0.38) | 0.93 |  | -0.16(-0.78, 0.46) | 0.59 |  | -0.27(-0.85,0.30) | 0.30 |  | -0.14(-0.49, 0.20) | 0.37 |
|  | Q4 | NA | NA |  | -0.13(-0.70, 0.44) | 0.60 |  | 0.57(0.10,1.04) | 0.02 |  | 0.1(-0.38, 0.59) | 0.65 |  | -0.25(-0.91,0.41) | 0.41 |  | 0.15(-0.32, 0.61) | 0.49 |

Notes: Children (6–11 years), adolescents (12–19 years) and adult (>19 years). Estimates were presented as standardized coefficients and 95% confidence intervals (CIs) and were adjusted for age (continuous), race/ethnicity (categorical), education level (categorical), body mass index (BMI) category (categorical), poverty income ratio (PIR, continuous), cotinine (categorical), the time of sample collection (categorical), exposure of bisphenol A, phthalates and polychlorinated biphenyls. Q1, Q2, Q3 and Q4 represent 1st to 4th quartiles of log2-HbEO. Abbreviations: TT, total testosterone; E2, total estradiol; SHBG, sex hormone binding globulin; FAI, free androgen index, was calculated as TT (ng /dL)/SHBG (nmol/L), TT/E2 was calculated as TT (ng /dL)/E2 (pm/ml).; HbEO, hemoglobin adducts of ethylene oxide;

**Supplementary Table S11.** Associations of continuous and quartiles of log2-HbEO with sex hormones by sex-puberty status in NHANES 2013–2016 adjusted exposure of bisphenol A, phthalates and polychlorinated biphenyls additionally.

|  | | Male | | | | | | | | | |  | | Female | | | | | | | | | |
| --- | --- | --- | --- | --- | --- | --- | --- | --- | --- | --- | --- | --- | --- | --- | --- | --- | --- | --- | --- | --- | --- | --- | --- |
|  | | | Prepubertal | | | |  | Pubertal | | | | |  | Prepubertal | | | Prepubertal | | | | | | |
|  | | β(95%CI) | | P-value |  | | | | β(95%CI) | P-value | | |  | β(95%CI) | | P-value | |  | | β(95%CI) | P-value | | |
| TT | Continuous log2-HbEO | -0.09(-0.49, 0.32) | | 0.66 |  | 0.04(-0.09, 0.17) | | | | | 0.47 | | |  | -0.1(-0.40,0.21) | 0.50 | |  | 0.01(-0.15, 0.18) | | | 0.83 |  |
|  | Q1 | ref | | ref |  | ref | | | | | ref | | |  | ref | ref | |  | ref | | | ref |  |
|  | Q2 | -0.26(-0.83, 0.31) | | 0.34 |  | -0.12(-0.42, 0.19) | | | | | 0.37 | | |  | -0.2(-0.63,0.22) | 0.32 | |  | -0.12(-0.42, 0.19) | | | 0.37 |  |
|  | Q3 | -0.26(-0.94, 0.42) | | 0.42 |  | 0.05(-0.22, 0.32) | | | | | 0.65 | | |  | 0.03(-0.72,0.77) | 0.94 | |  | 0.05(-0.22, 0.32) | | | 0.65 |  |
|  | Q4 | -0.02(-0.55, 0.50) | | 0.93 |  | 0.03(-0.34, 0.39) | | | | | 0.86 | | |  | -0.28(-0.78,0.22) | 0.24 | |  | 0.03(-0.34, 0.39) | | | 0.86 |  |
| E2 | Continuous log2-HbEO | NA | | NA |  | 0.13(-0.11,0.36) | | | | | 0.24 | | |  | 0.13(-0.05, 0.31) | 0.14 | |  | 0.13(-0.10, 0.35) | | | 0.22 |  |
|  | Q1 | NA | | NA |  | ref | | | | | ref | | |  | ref | ref | |  | ref | | | ref |  |
|  | Q2 | NA | | NA |  | 0.13(-0.26,0.51) | | | | | 0.44 | | |  | -0.12(-0.41, 0.18) | 0.41 | |  | -0.16(-0.76, 0.44) | | | 0.52 |  |
|  | Q3 | NA | | NA |  | -0.06(-0.40,0.28) | | | | | 0.66 | | |  | 0.22(-0.10, 0.54) | 0.16 | |  | 0.09(-0.54, 0.72) | | | 0.73 |  |
|  | Q4 | NA | | NA |  | 0.47(-0.28,1.22) | | | | | 0.17 | | |  | 0.13(-0.18, 0.45) | 0.38 | |  | 0.11(-0.49, 0.71) | | | 0.65 |  |
| SHBG | Continuous log2-HbEO | -0.11(-0.40, 0.19) | | 0.45 |  | 0.3( 0.06, 0.54) | | | | | **0.02** | | |  | -0.12(-0.33, 0.09) | 0.25 | |  | -0.16(-0.30,-0.03) | | | **0.02** |  |
|  | Q1 | ref | | ref |  | ref | | | | | ref | | |  | ref | ref | |  | ref | | | ref |  |
|  | Q2 | 0.32(-0.07, 0.72) | | 0.10 |  | -0.03(-0.55, 0.48) | | | | | 0.88 | | |  | -0.21(-0.61, 0.20) | 0.29 | |  | -0.14(-0.55, 0.27) | | | 0.44 |  |
|  | Q3 | 0.05(-0.25, 0.36) | | 0.72 |  | -0.08(-0.48, 0.32) | | | | | 0.65 | | |  | -0.24(-0.70, 0.21) | 0.27 | |  | -0.14(-0.48, 0.20) | | | 0.34 |  |
|  | Q4 | -0.28(-0.85, 0.29) | | 0.31 |  | 0.29(-0.07, 0.65) | | | | | 0.10 | | |  | -0.22(-0.65, 0.20) | 0.28 | |  | -0.35(-0.78, 0.09) | | | 0.10 |  |
| FAI | Continuous log2-HbEO | 0.03(-0.57,0.62) | | 0.93 |  | -0.25(-0.47,-0.02) | | | | | **0.04** | | |  | 0.03(-0.33,0.39) | 0.86 | |  | 0.16(-0.04, 0.37) | | | 0.10 |  |
|  | Q1 | ref | | ref |  | ref | | | | | ref | | |  | ref | ref | |  | ref | | | ref |  |
|  | Q2 | -0.59(-1.34,0.15) | | 0.11 |  | -0.09(-0.69,0.52) | | | | | 0.74 | | |  | 0.02(-0.45,0.49) | 0.93 | |  | -0.01(-0.47, 0.45) | | | 0.96 |  |
|  | Q3 | -0.31(-1.04,0.41) | | 0.37 |  | 0.12(-0.38,0.63) | | | | | 0.57 | | |  | 0.29(-0.58,1.16) | 0.48 | |  | 0.06(-0.34, 0.46) | | | 0.72 |  |
|  | Q4 | 0.26(-0.56,1.09) | | 0.51 |  | -0.25(-0.66,0.17) | | | | | 0.20 | | |  | -0.04(-0.67,0.60) | 0.90 | |  | 0.29(-0.30, 0.89) | | | 0.28 |  |
| TT/E2 | Continuous log2-HbEO | NA | | NA |  | -0.06(-0.22, 0.10) | | | | | 0.43 | | |  | -0.24(-0.58,0.10) | 0.16 | |  | -0.12(-0.31,0.06) | | | 0.17 |  |
|  | Q1 | NA | | NA |  | ref | | | | | ref | | |  | ref | ref | |  | ref | | | ref |  |
|  | Q2 | NA | | NA |  | -0.25(-0.60, 0.11) | | | | | 0.14 | | |  | -0.11(-0.51,0.30) | 0.58 | |  | 0.01(-0.50,0.53) | | | 0.96 |  |
|  | Q3 | NA | | NA |  | 0.1(-0.12, 0.33) | | | | | 0.30 | | |  | -0.22(-0.86,0.43) | 0.48 | |  | -0.17(-0.72,0.38) | | | 0.47 |  |
|  | Q4 | NA | | NA |  | -0.42(-0.98, 0.14) | | | | | 0.11 | | |  | -0.44(-0.98,0.10) | 0.10 | |  | -0.17(-0.80,0.46) | | | 0.53 |  |

Notes: Puberty status was defined as “pubertal” if TT ≥ 50 ng/dL in males, E2 ≥ 20 pg/ml or menstrual period started in females, otherwise puberty status was defined as “prepubertal”. Estimates were presented as standardized coefficients and 95% confidence intervals (CIs) and were adjusted for age (continuous), race/ethnicity (categorical), education level (categorical), body mass index (BMI) category (categorical), poverty income ratio (PIR, continuous), cotinine (categorical), the time of sample collection (categorical), exposure of bisphenol A, phthalates and polychlorinated biphenyls. Analyses for E2 were not performed in prepubertal participants as the detection frequency of E2 < 50% in that group. Q1, Q2, Q3 and Q4 represent 1st to 4th quartiles of log2-HbEO. “NA” indicates the estimates were not available in prepubertal participants as the detection frequency of E2 was < 50% in that group. Abbreviations: TT, total testosterone; E2, total estradiol; SHBG, sex hormone binding globulin; FAI, free androgen index, was calculated as TT (ng /dL)/SHBG (nmol/L), TT/E2 was calculated as TT (ng /dL)/E2 (pm/ml); HbEO, hemoglobin adducts of ethylene oxide;

**Supplementary Table S12.** Associations of continuous and quartiles of log2-HbEO with sex hormones in adults in NHANES 2013–2016 after excluded participants aged ≥65 years.

| **Sex hormones** |  | **Male (adult)** | |  | **Female (adult)** | |
| --- | --- | --- | --- | --- | --- | --- |
|  |  | **β(95%CI)** | **P-value** |  | **β(95%CI)** | **P-value** |
| **TT** | Continuous log2-HbEO | -0.15(-0.64, 0.34) | 0.53 |  | 0.26(0.04, 0.48) | **0.03** |
|  | Q1 | ref | ref |  | ref | ref |
|  | Q2 | -0.54(-1.23,0.15) | 0.12 |  | 0.11(-0.34, 0.57) | 0.56 |
|  | Q3 | -0.37(-1.02,0.28) | 0.24 |  | 0.14(-0.32, 0.59) | 0.50 |
|  | Q4 | -0.46(-1.26,0.34) | 0.24 |  | 0.38(-0.13, 0.90) | 0.12 |
| **E2** | Continuous log2-HbEO | NA | NA |  | 0.17(-0.04,0.39) | 0.10 |
|  | Q1 | NA | NA |  | ref | ref |
|  | Q2 | NA | NA |  | 0.14(-0.23,0.50) | 0.39 |
|  | Q3 | NA | NA |  | -0.07(-0.39,0.26) | 0.62 |
|  | Q4 | NA | NA |  | 0.55(-0.11,1.22) | 0.09 |
| **SHBG** | Continuous log2-HbEO | -0.12(-0.45, 0.20) | 0.43 |  | 0.24(0.01, 0.46) | **0.04** |
|  | Q1 | ref | ref |  | ref | ref |
|  | Q2 | 0.41(0.02, 0.79) | 0.04 |  | 0(-0.38, 0.39) | 0.98 |
|  | Q3 | 0.15(-0.15, 0.46) | 0.29 |  | 0.13(-0.21, 0.47) | 0.40 |
|  | Q4 | -0.23(-0.80, 0.33) | 0.39 |  | 0.43(-0.10, 0.95) | 0.09 |
| **FAI** | Continuous log2-HbEO | -0.02(-0.76, 0.72) | 0.96 |  | 0.02(-0.28,0.33) | 0.87 |
|  | Q1 | ref | ref |  | ref | ref |
|  | Q2 | -0.96(-1.82, -0.11) | 0.03 |  | 0.11(-0.44,0.66) | 0.65 |
|  | Q3 | -0.53(-1.30, 0.25) | 0.17 |  | 0.01(-0.54,0.56) | 0.96 |
|  | Q4 | -0.22(-1.33, 0.90) | 0.69 |  | -0.03(-0.62,0.57) | 0.92 |
| **TT/E2** | Continuous log2-HbEO | NA | NA |  | 0.1(-0.09, 0.30) | 0.27 |
|  | Q1 | NA | NA |  | ref | ref |
|  | Q2 | NA | NA |  | -0.02(-0.41, 0.37) | 0.91 |
|  | Q3 | NA | NA |  | 0.22(-0.14, 0.57) | 0.19 |
|  | Q4 | NA | NA |  | -0.13(-0.70, 0.44) | 0.60 |

Notes: Estimates were presented as standardized coefficients and 95% confidence intervals (CIs) and were adjusted for age (continuous), race/ethnicity (categorical), education level (categorical), body mass index (BMI) category (categorical), poverty income ratio (PIR, continuous), cotinine (categorical), the time of sample collection (categorical), energy intake (categorical), physical activity (categorical), and prescription medication (categorical). Q1, Q2, Q3 and Q4 represent 1st to 4th quartiles of log2-HbEO. Abbreviations: TT, total testosterone; E2, total estradiol; SHBG, sex hormone binding globulin; FAI, free androgen index, was calculated as TT(ng /dL)/SHBG (nmol/L), TT/E2 was calculated as TT (ng /dL)/E2 (pm/ml); HbEO, hemoglobin adducts of ethylene oxide;
